# Site-selective covalent functionalization at interior carbon atoms and on the rim of circumtrindene, a C_36_H_12_ open geodesic polyarene

**DOI:** 10.3762/bjoc.10.94

**Published:** 2014-04-28

**Authors:** Hee Yeon Cho, Ronald B M Ansems, Lawrence T Scott

**Affiliations:** 1 Department of Chemistry, Merkert Chemistry Center, Boston College, Chestnut Hill, Massachusetts 02467-3860, USA

**Keywords:** Bingel–Hirsch reaction, buckybowl, carbon nanomaterials, cyclopropanation, polycyclic aromatic hydrocarbon, Prato reaction

## Abstract

Circumtrindene (**6**, C_36_H_12_), one of the largest open geodesic polyarenes ever reported, exhibits fullerene-like reactivity at its interior carbon atoms, whereas its edge carbons react like those of planar polycyclic aromatic hydrocarbons (PAHs). The Bingel–Hirsch and Prato reactions – two traditional methods for fullerene functionalization – afford derivatives of circumtrindene with one of the interior 6:6 C=C bonds modified. On the other hand, functionalization on the rim of circumtrindene can be achieved by normal electrophilic aromatic substitution, the most common reaction of planar PAHs. This peripheral functionalization has been used to extend the π-system of the polyarene by subsequent coupling reactions and to probe the magnetic environment of the concave/convex space around the hydrocarbon bowl. For both classes of functionalization, computational results are reported to complement the experimental observations.

## Introduction

**Figure 1:**
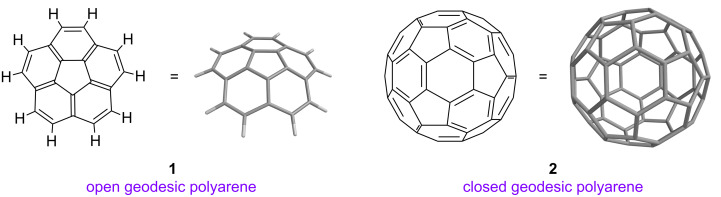
Prototypical open and closed geodesic polyarenes.

Investigations into the structures and properties of geodesic polyarenes began with the synthesis of corannulene (**1**, C_20_H_10_) in 1966 [1–2] and were greatly stimulated by the discovery of buckminsterfullerene (**2**, C_60_) in 1985 (Figure 1) [3]. Whereas fullerenes comprising complete three-dimensional polyhedra can be classified as “closed” geodesic polyarenes, curved subunits of fullerenes are regarded as “open” geodesic polyarenes [4]. The fullerenes constitute one family of geodesic polyarenes, and bowl-shaped polycyclic aromatic hydrocarbons (PAHs) constitute another.

**Figure 2:**
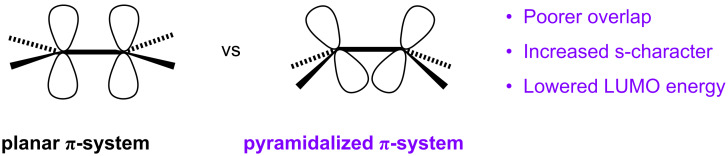
Planar vs pyramidalized π-system.

Both open and closed geodesic polyarenes are characterized by curved π-systems composed of pyramidalized carbon atoms (Figure 2). Pyramidalization not only imposes curvature, but it also imparts significant electronic perturbations. The less parallel the alignment of p-orbitals, for example, the poorer will be their overlap, and that will lead to weaker π-bonds. Weakening a π-bond raises the energy of the bonding π molecular orbital (MO) and lowers the energy of the antibonding π*-MO. This is one important consequence of pyramidalization. A second consequence is the mixing of s-orbital character with the p-orbitals in the π-system. This hybridization lowers the energy of each atomic orbital in the π-system and thereby lowers the energy of all the derived π-MOs, i.e., both the HOMO (highest occupied molecular orbital) and the LUMO (lowest unoccupied molecular orbital) [5]. This lowering of the HOMO offsets the raising of the HOMO caused by poorer π overlap; however, lowering the LUMO as a consequence of orbital hybridization amplifies the lowering caused by the weaker π-bond. The major electronic consequence of π-system curvature, therefore, is a substantial lowering of the energy of the LUMO, while leaving the energy or the HOMO relatively unchanged.

Considerable effort by a number of research groups has been devoted to the preparation of fullerene fragments and to studies of their chemical and physical properties [6–9]. Several laboratories, including the authors’, have been particularly successful in synthesizing geodesic polyarenes using flash vacuum pyrolysis (FVP) [6]. The FVP method involves slow sublimation of a starting material under vacuum, rapid passage of the gas-phase molecules through a hot zone, and subsequent capture of the products in a cold trap [10–12]. The high temperatures used in FVP (often up to 1100 °C) provide sufficient thermal energy to enable molecules to overcome high activation energy barriers.

**Figure 3:**
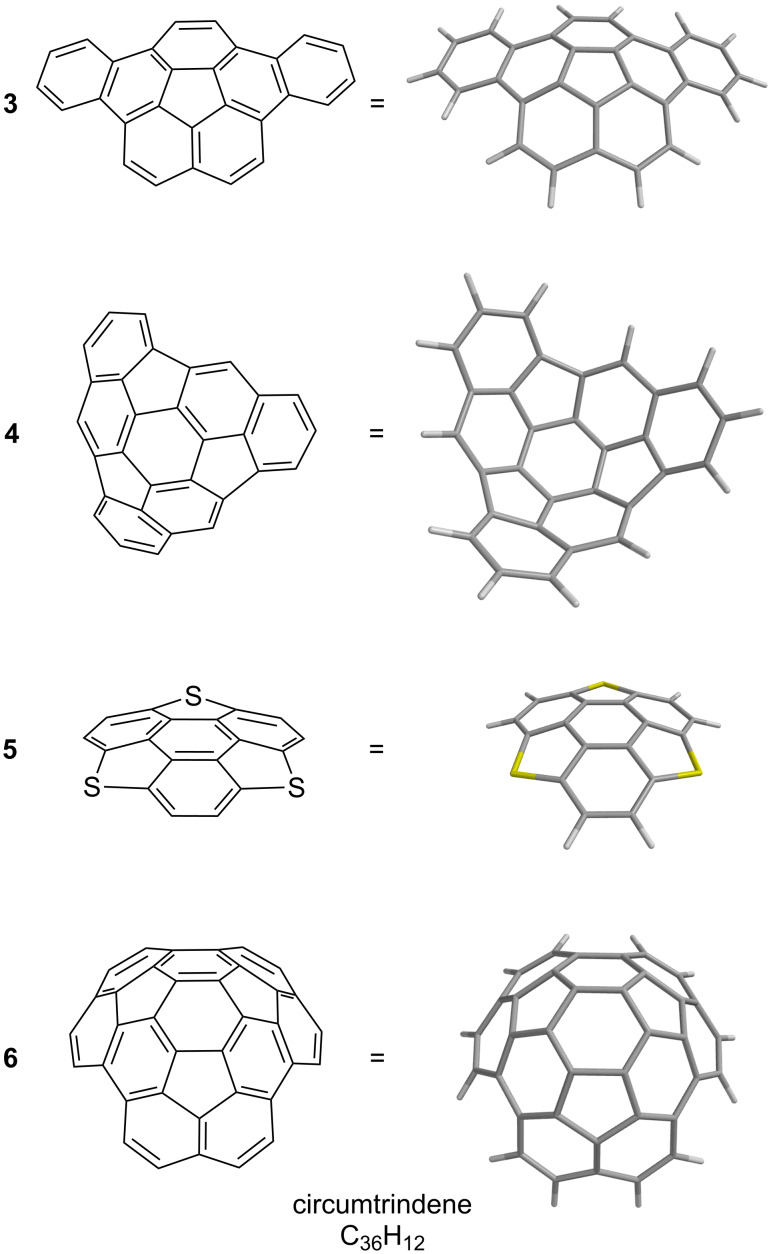
Selected examples of geodesic polyarenes synthesized by FVP.

The FVP approach takes advantage of the fact that the normal out-of-plane deformations of simple planar polyarenes become greatly amplified at high temperatures. FVP has been employed to prepare numerous highly-curved polyarenes, including corannulene (**1**) [[13–14], dibenzo[*a*,*g*]corannulene (**3**, C_28_H_14_) [15], a *C*_3_-symmetric hemifullerene (**4**, C_30_H_12_) [16–17], the geodesic heterocycle triphenyleno[*1,12-bcd:4,5-b’c’d’:8,9-b’’c’’d’’*]trithiophene (**5**, C_18_H_6_S_3_) [18], the deep buckybowl circumtrindene (**6**, C_36_H_12_) [19–20], and even fullerene C_60_ (**2**) (Figure 1 and Figure 3) [21–22]. Syntheses of curved PAHs are not limited, however, to FVP; non-pyrolytic, “wet chemical” methods to access fullerene fragments have also been developed in our laboratory and elsewhere [1–2,15,23–27].

**Scheme 1:**
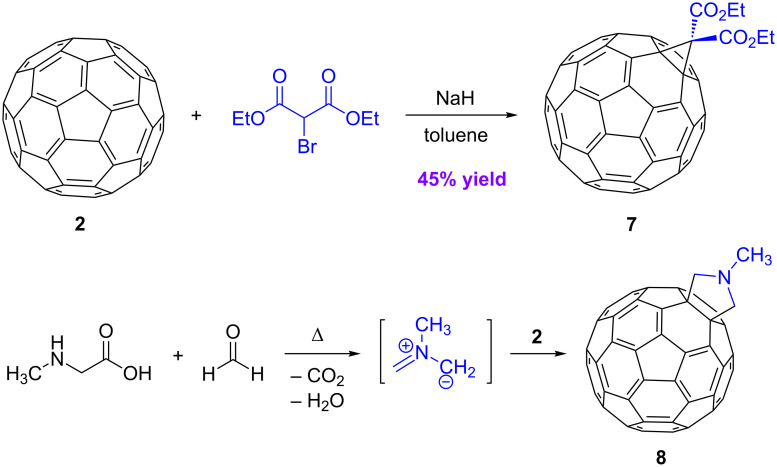
Covalent functionalization of fullerene C_60_ by the Bingel–Hirsch reaction and the Prato reaction.

In addition to synthesis, the functionalization of geodesic polyarenes – by both covalent and noncovalent bonding – has attracted significant attention in recent years. The functionalization of fullerenes was intensively explored immediately following the discovery of methods for bulk preparation of fullerenes in 1990 [28]. Within the first few years, several covalent functionalization sequences were introduced that became widely used for the construction of multifunctional architectures with C_60_ as an integral building unit. Representative synthetic reactions of fullerene C_60_ (**2**) include cyclopropanation [29–30], [3 + 2] cycloaddition [31–32], [4 + 2] cycloaddition [33], nucleophilic addition [34], and radical addition reactions [35]. Two of the earliest and most widely used reactions, the Bingel–Hirsch reaction [29–30] and the Prato reaction [31–32] are illustrated in Scheme 1. The Bingel–Hirsch reaction affords a cyclopropanated fullerene **7**, and the Prato reaction gives a [3 + 2] cycloaddition adduct **8**. Concurrent with the development of covalent functionalization, numerous noncovalent functionalization methods were also investigated. In particular, the preparation of endohedral fullerenes, having metal atoms or other “guests” trapped inside the fullerene cage, opened yet another new branch of fullerene chemistry [36–37].

**Scheme 2:**
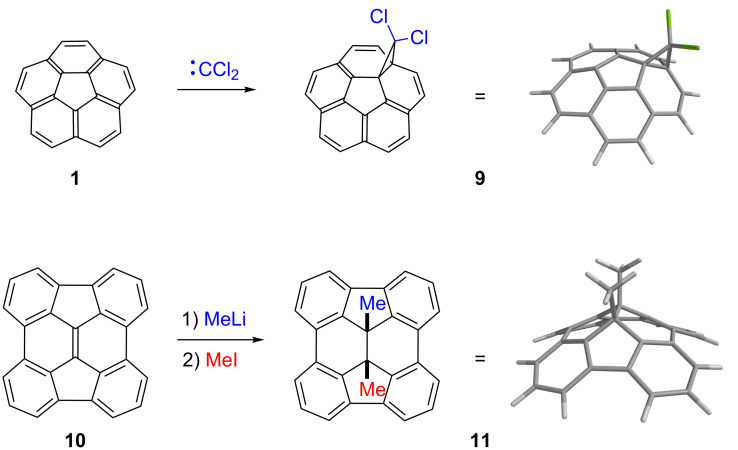
Fullerene-type chemistry at interior carbon atoms of corannulene (**1**) and diindenochrysene (**10**).

Our laboratory has been interested in the covalent functionalization of open geodesic polyarenes at their interior carbon atoms (i.e., carbon atoms that are shared by three rings) [4,38]. The curved PAHs corannulene (**1**) and diindenochrysene (**10**), for example, show fullerene-type reactivity towards carbenes and nucleophiles, respectively (Scheme 2). Dichlorocarbene adds to corannulene (**1**) to afford predominantly the cyclopropanated product **9** [39], whereas methyllithium adds to **10**, giving the 1,2-addition product **11**, after alkylation of the resulting anion by methyl iodide [40]. In contrast to these examples, no planar PAH has ever been observed to suffer direct covalent bond formation at an interior carbon atom, despite the long history of aromatic hydrocarbon chemistry [41–42].

**Figure 4:**
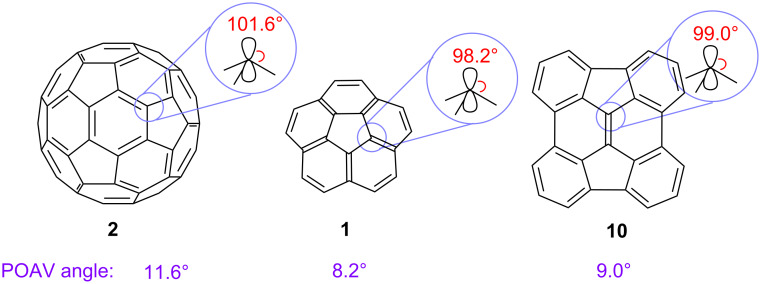
POAV angles of fullerene C_60_ (**2**), corannulene (**1**), and diindenochrysene (**10**).

These PAHs (**1** and **10**) are obviously nonplanar and have the same patterns of pentagons and hexagons as that found on the surface of fullerene C_60_. The distortions of the π-systems in these molecules, however, are less severe than that in C_60_, as indicated by their π-orbital axis vector (POAV) angles [43–44]. The POAV angles of C_60_ (**2**), corannulene (**1**), and diindenochrysene (**10**) are 11.6° (101.6 –90° = 11.6° pyramidalization), 8.2°, and 9.0°, respectively (Figure 4) [23–24,45]. Furthermore, the barrier for bowl-to-bowl inversion in corannulene (10.2 kcal/mol at −64 °C) [46] is so low that corannulene inverts rapidly even at room temperature. The inversion barrier of diindenochrysene (**10**) was calculated to be even lower (6.7 kcal/mol at ambient temperature by B3LYP/6-311G**) [47].

While there are many examples of covalent chemistry for fullerenes (i.e., closed geodesic polyarenes), covalent functionalizations of open geodesic polyarenes other than corannulene have been reported only rarely. As in fullerene chemistry, chemical derivatization of buckybowls can be expected to change the electronic, optical, magnetic, mechanical, and chemical properties of the material. For such functionalization reactions, site selectivity often becomes an issue when there is more than one site where the reaction could occur. Herein, we report methods for the site-selective covalent functionalization of circumtrindene (**6**), one of the most highly-curved fullerene fragments known, comprising 60% of the C_60_ ball.

## Results and Discussion

### Synthesis and properties of circumtrindene (**6**)

**Scheme 3:**
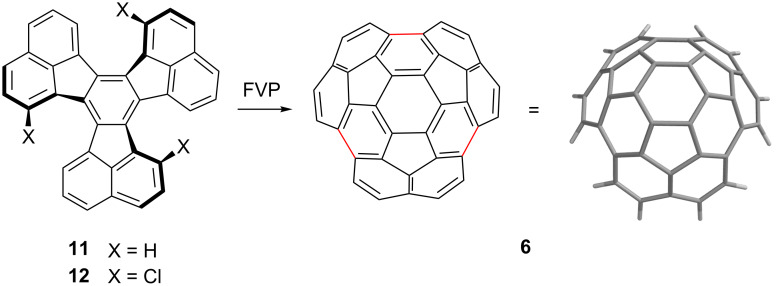
Synthesis of circumtrindene (**6**) by FVP.

In 1996, our laboratory reported that the C_36_H_12_ bowl circumtrindene (**6**) could be obtained by flash vacuum pyrolysis of decacyclene (**11**, Scheme 3) [19]. Extreme temperatures, in the range of 1200–1300 °C, were required, and the yield was less than 1%, but the transformation gave 60% of the C_60_ ball in multimilligram quantities in a single step from a cheap, commercially available starting material with no reagents other than heat. Obviously, this brute-force triple-cyclodehydrogenation is not an efficient process. The yield could be improved dramatically, however, by the incorporation of substituents capable of generating aryl radicals by C–X bond homolysis in the fjord regions; the C_36_H_12_ bowl was produced in 26% yield from 3,9,15-trichlorodecacyclene (**12**) by FVP at 1100 °C (Scheme 3) [20].

The importance of strategically functionalizing hydrocarbon precursors in a way that will generate radical centers where new bonds are to be formed by FVP is dramatically illustrated by the vastly improved efficiency of this triple cyclization reaction when starting from **12** instead of **11**. Unfortunately, the extra mass and polarity introduced by the chlorine atoms impedes sublimation of the substrate. A thin stream of nitrogen carrier gas leaked into the sample chamber largely overcomes this complication; however, even with this modification of the set-up, some 30% of starting material **12** decomposes in the sample chamber and fails to sublime.

**Scheme 4:**
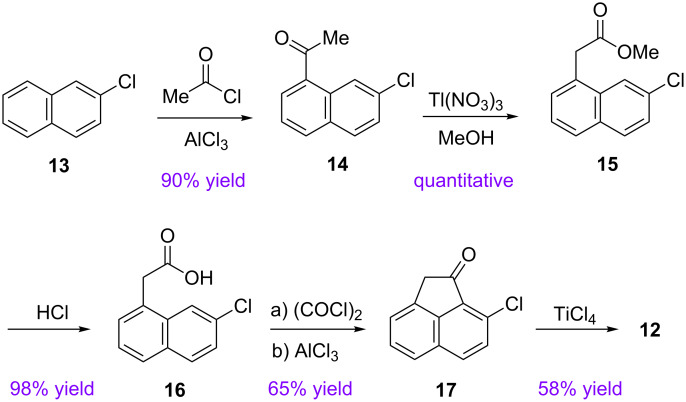
Synthetic route to 3,9,15-trichlorodecacyclene (**12**).

The halogenated FVP precursor, 3,9,15-trichlorodecacyclene (**12**), was prepared in five steps from commercially available starting materials, following the same route reported in our earlier publication (Scheme 4) [20]. Cyclotrimerization of **17** to the *C*_3_-symmetric trichlorodecacyclene **12** was accomplished by a triple aldol condensation of the chloro ketone **17** [48–49]. Improvements in the procedures reported previously are described in the experimental section in the Supporting Information File 1.

**Figure 5:**
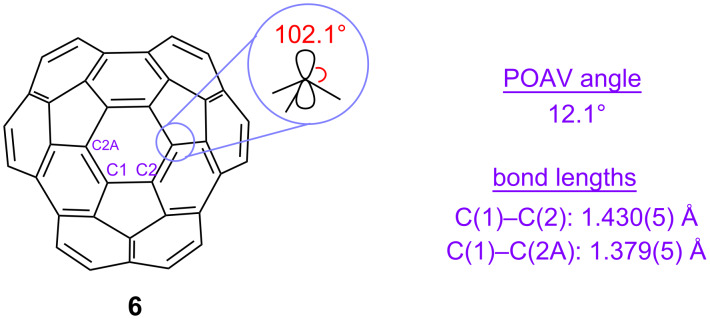
POAV angle and bond lengths of circumtrindene.

In agreement with DFT calculations [50] the X-ray crystal structure of circumtrindene (**6**) confirms that the carbon atoms comprising the top of the dome are distorted from planarity to an even greater degree than the carbon atoms in C_60_ [51]; the pyramidalization in circumtrindene (POAV angle = 12.1° [52], Figure 5) significantly surpasses that in fullerene C_60_ (POAV angle = 11.6°, Figure 4). Moreover, the X-ray structure of circumtrindene (**6**) reveals that the carbon–carbon bonds around the 6-membered ring at the top of the dome are not equivalent. The bond lengths vary depending on their location (Figure 5), as is also observed in fullerenes [53]. The carbon–carbon bond shared by two six-membered rings (so-called 6:6-bonds: 1.379 Å) are shorter than those shared by a 5-membered ring and a 6-membered ring (5:6-bonds: 1.430 Å). That is, the carbon–carbon bonds of the hexagonal ring in the center of circumtrindene are not the same as those in benzene. They look more like those of a “cyclohexatriene” than as those of a benzene ring.

The ^1^H NMR spectrum of circumtrindene (**6**) consists of two doublets at δ 7.58 and 7.20 ppm. Induced diamagnetic ring currents in this conjugated π-system deshield the hydrogen atoms on the rim; however, the magnitude of the effect is significantly less pronounced than that seen even in smaller PAHs that are less curved or planar [54]. Clearly, the high degree of curvature in this geodesic polyarene has a strong impact on the NMR resonances. The unequal magnitudes of the ring currents on the convex and concave faces of circumtrindene are probed and discussed in a later section.

### Interior functionalization of circumtrindene

Unlike planar PAHs, circumtrindene (**6**) exhibits fullerene-like covalent chemistry at its “interior” carbon atoms. Two examples are reported below [55].

#### Bingel–Hirsch reaction

The Bingel–Hirsch reaction produces 3-membered rings by a reaction that begins with a nucleophilic addition to a fullerene (Scheme 1). It is considered to be one of the most valuable preparative methods for functionalizing fullerenes, since it changes both the solubility and the electrochemical behavior.

**Scheme 5:**
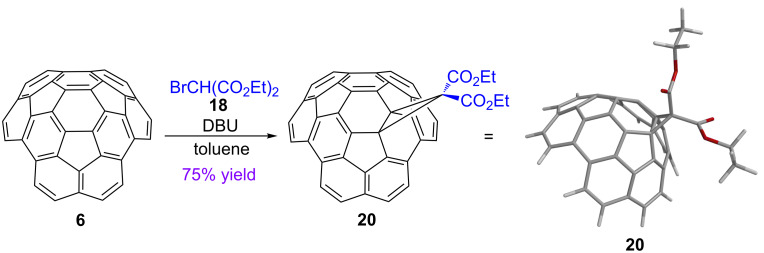
Bingel–Hirsch reaction of circumtrindene (**6**).

Considering the similarity between the curved π-systems of circumtrindene and fullerene C_60_, we speculated that the Bingel–Hirsch reaction might occur with circumtrindene (**6**) in the same manner as it does with fullerene C_60_. Gratifyingly, the cyclopropanation of circumtrindene by the Bingel–Hirsch reaction proceeds smoothly to afford **20** in good yield and with complete site selectivity (Scheme 5). The reaction was performed with five equivalents of bromomalonate and one equivalent of DBU (1,8-diazabicyclo[5.4.0]undec-7-ene) as a base in toluene at room temperature.

**Scheme 6:**
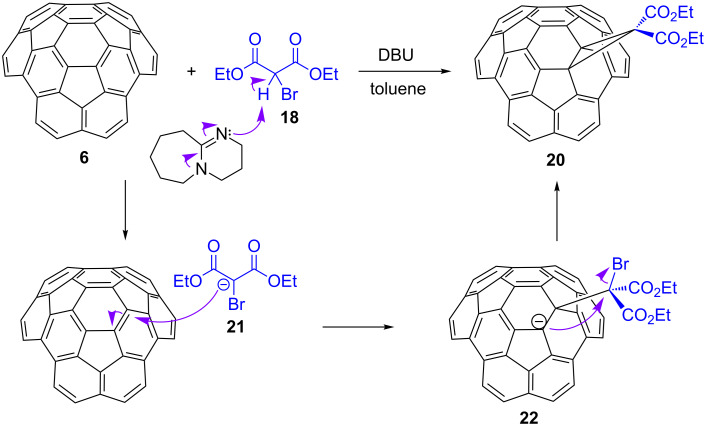
Proposed mechanism for the Bingel–Hirsch reaction of circumtrindene (**6**).

Based on mechanism studies on the corresponding reaction of fullerenes, the mechanism in Scheme 6 is proposed for the Bingel–Hirsch reaction of circumtrindene. The enolate generated by deprotonation of bromomalonate **18** with DBU functions as a nucleophile; it attacks one of the strongly pyramidalized carbon atoms of circumtrindene, and subsequent intramolecular displacement of the bromide generates the cyclopropanated circumtrindene **20**. As with fullerenes, this cyclopropanation reaction occurs exclusively at a 6:6-bond of circumtrindene.

In contrast to the highly symmetrical fullerene C_60_, circumtrindene has three distinct sets of 6:6-bonds. Only structure **20**, however, is consistent with the ^1^H and ^13^C NMR spectra of the cyclopropanated circumtrindene obtained (*C*_s_-symmetric with non-equivalent esters, see Supporting Information File 1). Thus, the reaction is site selective for the 6:6-bond at the point of greatest curvature on the convex surface (i.e., the carbon atoms with the greatest POAV angle, see Figure 5). Similar site selectivity for the 6:6-bond that incorporates the most pyramidalized carbon atoms has previously been observed in the functionalization of fullerene C_70_ [56].

As explained in the introductory section, curving a π-system lowers the energy of the LUMO as a consequence of both poorer π overlap and increased s-character in the atomic orbitals used for π-bonding. The unusually high reactivity of both fullerenes and circumtrindene towards nucleophiles is one manifestation of these lowered LUMO energies. In the initial nucleophilic attack (Scheme 6), the nucleophile adds to the site with the largest LUMO coefficient (see below for DFT calculations on the LUMO of circumtrindene).

This reaction also proceeds with excellent facial selectivity; the cyclopropane ring is formed exclusively on the convex face of circumtrindene. The different electron densities of the two faces reinforce the steric bias for addition to the convex face. As a consequence of the curved π-system, the convex face has less electron density than the concave face (see DFT calculations below), and that renders the convex side electronically more favorable for a nucleophilic attack.

#### Prato reaction

Cycloadditions to the π-system constitute another major family of fullerene reactions. In particular, 1,3-dipolar reagents readily add to the 6:6-bond of fullerene C_60_, which acts as a good dipolarophile. Among such [3 + 2] cycloadditions, the reaction with azomethine ylides has found great popularity, because it leads to versatile pyrrolidine derivatives of C_60_. The sources of the ylide can be iminium salts, aziridines, oxazolidines or silylated iminium compounds. In 1993, Prato and coworkers developed the protocol that is now used most commonly [31–32]. In the first step, an *N-*substituted amino acid (e.g., *N*-methylglycine) reacts with an aldehyde or ketone to generate an azomethine ylides in situ. Trapping of the ylide by a fullerene provides a fullerene-pyrrolidine derivative (**8**), as illustrated in Scheme 1.

**Scheme 7:**
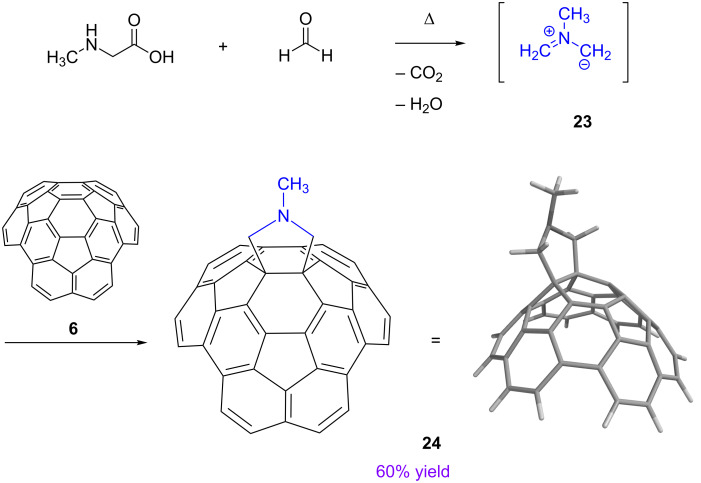
Prato reaction of circumtrindene (**6**).

In light of the great electrophilicity of circumtrindene in the Bingel–Hirsch reaction, it came as no surprise that circumtrindene can act as a good dipolarophile in a [3 + 2] cycloaddition reaction as well. Accordingly, the azomethine ylide **23** generated in situ from *N*-methylglycine and formaldehyde adds to circumtrindene in refluxing benzene to give the pyrrolidine derivative **24** (Scheme 7). As in the Bingel–Hirsch reaction, the 1,3-dipole reacts exclusively with the 6:6-bond in circumtrindene that contains the most pyramidalized carbon atoms in the molecule.

Considering the [3 + 2] cycloaddition processes in terms of frontier molecular orbitals, the dominant interaction should be that between the HOMO of the 1,3-dipole and the LUMO of the dipolarophile. In the Prato reaction in Scheme 7, the electron-rich ylide seeks out the carbon atoms of circumtrindene with the highest LUMO coefficient. As in the Bingel–Hirsch reaction above, the Prato reaction of circumtrindene likewise shows complete facial selectivity for the convex face.

#### Molecular orbitals and electrostatic potentials

Both of these fullerene-type functionalization reactions at the interior carbon atoms of circumtrindene show complete site and facial selectivity. The molecule behaves as an electrophile toward the bromoenolate (deprotonated bromomalonate) in the Bingel–Hirsch reaction and as an electrophile toward the 1,3-dipole (azomethine ylide) in the Prato reaction. Consequently, the LUMO of circumtrindene is the frontier molecular orbital that should control these interior functionalizations. As noted above, the distortion of the circumtrindene π-system imposed by its geodesic curvature is expected to lower the LUMO energy of the molecule substantially, thereby enhancing its reactivity (see Figure 2).

**Figure 6:**
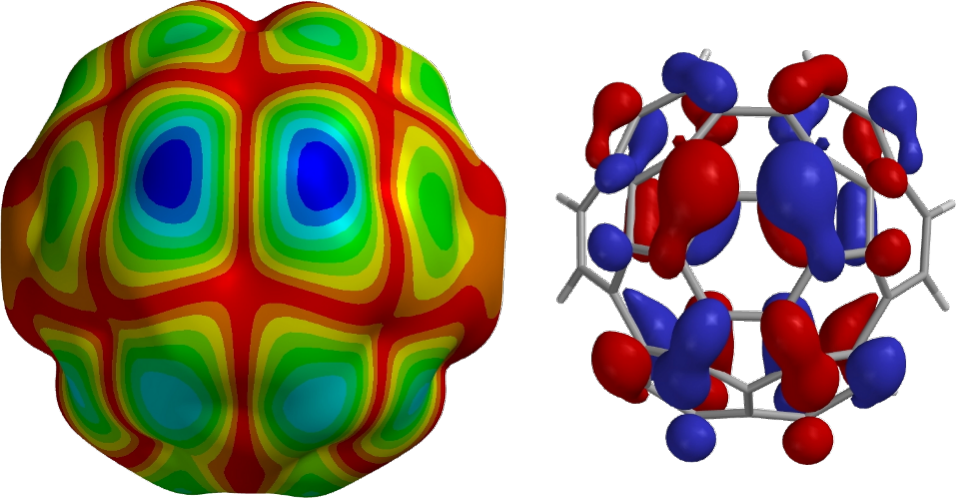
LUMO orbital map of circumtrindene (B3LYP/6-31G*). The darkest blue areas correspond to the regions with the largest LUMO coefficients.

We have calculated the LUMO of circumtrindene at the B3LPY/6-31G* level of theory, using the curved geometry obtained by optimization at the same level of theory, and find that it is doubly degenerated. Figure 6 depicts one of these antibonding orbitals in two different formats. As these images show, the LUMO of circumtrindene has its largest coefficients at the interior 6:6-bond composed of carbon atoms with the greatest degree of pyramidalization. This is precisely where the Bingel–Hirsch reaction and the Prato reaction of circumtrindene occur. The DFT calculations and the experimental results are therefore in complete agreement.

**Figure 7:**
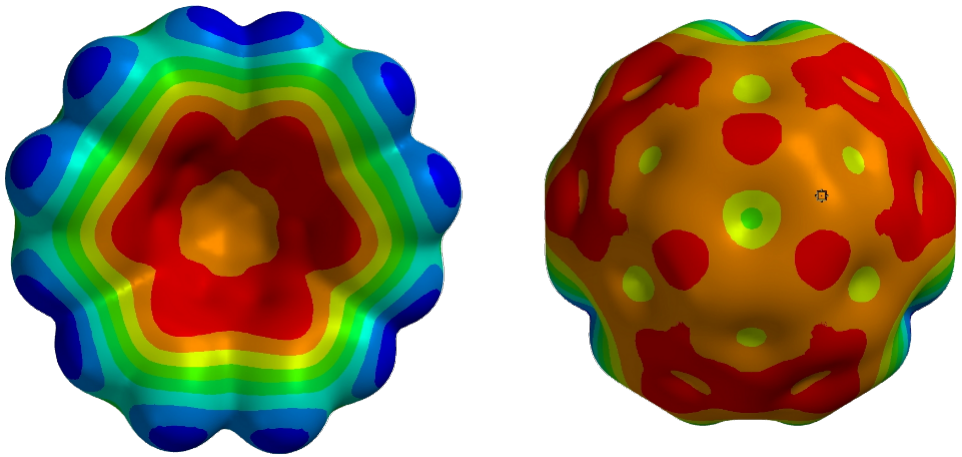
Electrostatic potentials on the surfaces of circumtrindene (B3LPY/6-31G*).

As another consequence of π-system curvature, the electron densities for the two different faces of circumtrindene are expected to be unequal [57]. Density functional calculations (B3LPY/6-31G*) reveal that the outer surface of circumtrindene should be relatively electron deficient, resembling that of C_60_, whereas the inside surface should be relatively electron rich (Figure 7). The high facial selectivity of these interior functionalization reactions, giving exclusive covalent bond formation on the convex face with electron-rich reaction partners, is also consistent with the DFT calculations.

#### Peripheral functionalization of circumtrindene

Since the isolation and characterization of the first polycyclic aromatic hydrocarbons in the 1800s, a tremendous range of chemical reactions on such compounds has been developed [41–42]. Most of this chemistry involves chemical modifications on the edge carbons and has been used to functionalize the existing polyarenes and/or to extend them to larger PAHs. The most common class of reactions is the electrophilic aromatic substitution; however, free radical, nucleophilic addition, reduction, and oxidation reactions are also possible. To synthesize larger PAHs that are not available from natural sources or to make existing ones in more rational ways, functionalization reactions can be followed by functional group interconversions and coupling reactions.

**Figure 8:**
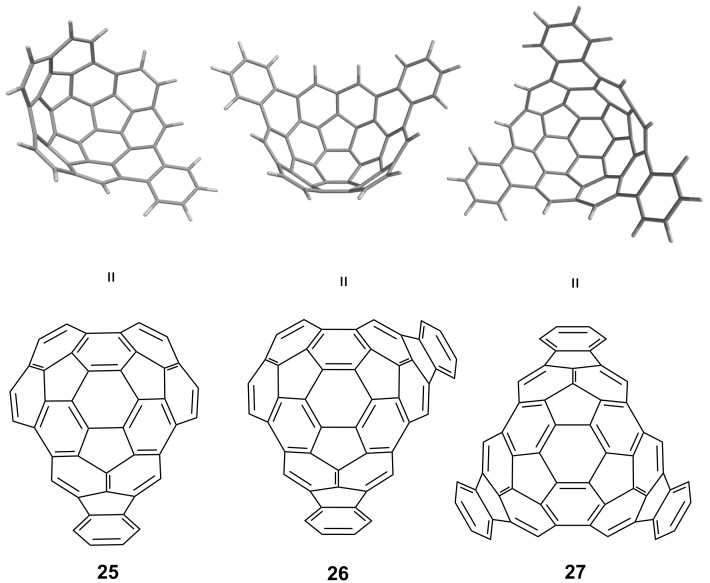
Monoindeno- (**25**), diindeno- (**26**), and triindenocircumtrindene (**27**).

As discussed above, the synthesis of bowl-shaped geodesic polyarenes is non-trivial, owing to the great strain energy in such molecules. In this regard, it is generally easier to construct deeper bowls from already existing curved molecules, rather than to make them from planar building blocks. Circumtrindene (**6**) has a curvature comparable to C_60_ fullerene and, therefore, has much of the strain already built in that is needed to synthesize deeper bowls. Circumtrindene represents 60% of the network of sp^2^ carbons that constitute C_60_ fullerene; to go to 70%, 80%, and 90% of C_60_, the number of carbon atoms added in each step would be six, as in monoindeno-, diindeno-, and triindenocircumtrindene (**25–27**) (Figure 8). We envisioned that circumtrindene might be used as a building block from which to prepare deeper bowls by a peripheral functionalization method with standard aromatic chemistry.

Such functionalization reactions will also allow us to probe the magnetic environments of the two different faces of circumtrindene. As illustrated in Figure 8, the inside surface of circumtrindene is considerably more electron rich than the outside surface. If a suitable substituent could be placed on the rim of circumtrindene that hangs probe groups over both the concave and the convex faces without restraints, it would be possible to explore the different magnetic environments above the two surfaces experimentally.

#### Ring extension

**Figure 9:**
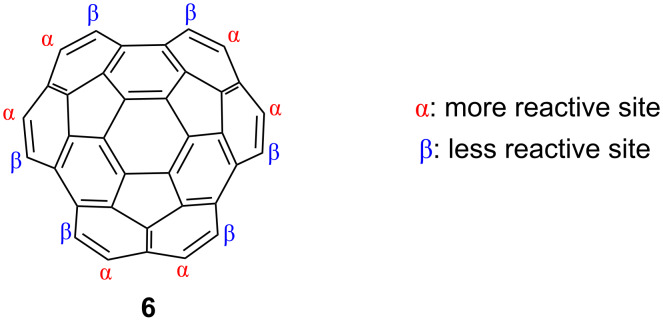
Two different types of rim carbon atoms on circumtrindene.

To extend the bowl and π-system of circumtrindene, a reasonable first step would be functionalizing the rim of circumtrindene with a group that is suitable for subsequent coupling reactions, e.g., a bromine atom (Figure 9). Since the α-positions of the naphthalene subunits should be more reactive than the crowded β-positions, the site selectivity between the two types of rim carbon atoms was not expected to present a problem.

**Scheme 8:**
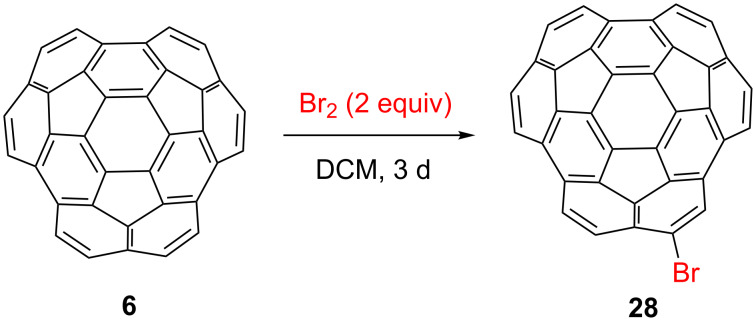
Site-selective peripheral monobromination of circumtrindene.

We were aware, however, that the interior carbon atoms of circumtrindene might also be brominated. Fullerenes are known to undergo bromine addition with elemental bromine in chlorinated solvents at room temperature [28]. To avoid the unwanted bromination at interior carbon atoms, the reaction conditions were carefully optimized (see Supporting Information File 1 for more details). In a reasonably dilute solution and with two equivalents of bromine, selective monobromination on the α-carbon of circumtrindene has been achieved with a quantitative yield (Scheme 8).

**Scheme 9:**
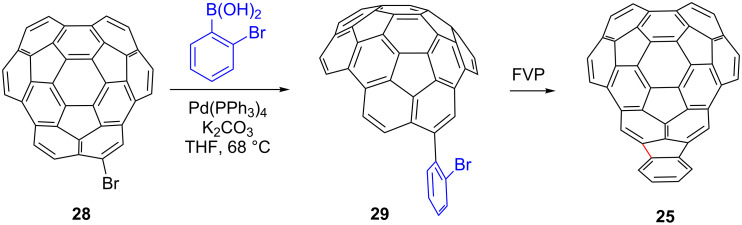
Suzuki coupling and ring-closing reactions toward indenocircumtrindene (**25**).

With bromocircumtrindene **28** in hand, Suzuki coupling reactions were carried out with commercially available boronic acids. The reaction proceeds smoothly with 2-bromophenylboronic acid in the presence of Pd(0) and a base to furnish **29** (Scheme 9). The Suzuki reaction proceeds reasonably well, despite the potential complication of the product undergoing a subsequent Suzuki coupling with the bromine of the phenyl group. The next step of the sequence, a ring closure to indenocircumtrindene (**25**), was accomplished by FVP. The bromine in the *ortho*-position of the phenyl group is presumably lost by homolytic bond cleavage, and the resulting aryl radical then cyclizes [6]. Unfortunately, the reaction also generates a significant amount of circumtrindene by loss of the phenyl group during the FVP process [58].

In an effort to improve on the yield over the FVP process, solution-phase ring-closure methods were also examined. All attempts to close **29** to indenocircumtrindene (**25**) by intramolecular arylations (Heck-type cyclization), unfortunately, were unsuccessful. Heck-type cyclizations work nicely with planar PAHs and even on moderately curved PAHs, such as corannulene [15,23–24]. Nonetheless, when applied to the indenocircumtrindene synthesis, the reaction did not give any ring-closed products. Oxidative addition of Pd(0) into one of the strained C–C bonds on the rim of circumtrindene might be competing with the desired cyclization [59]; however, we have not characterized any of the decomposition products from this reaction.

#### Magnetic environments

**Scheme 10:**
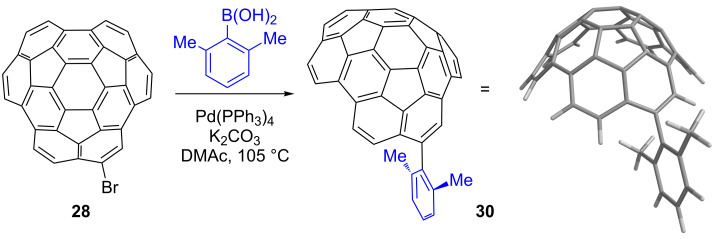
Suzuki coupling to prepare compound **30**.

Induced diamagnetic ring currents strongly shield nuclei that are positioned over the faces of aromatic rings. In planar PAHs, the π-orbitals are perpendicular to the σ-bonds, the electrons are distributed equally on the two faces of the aromatic system, and the ring currents on the two faces are equivalent. When curvature is built into molecules, however, the electron densities of the two faces become unequal, and the shielding ability of the ring currents are no longer expected to be the same [60]. To probe the ring currents of the two faces of circumtrindene, we designed a compound with groups hanging over both the concave and the convex faces. Such a compound can be synthesized by replacing the bromo group of **28** with a 2,6-dimethyl substituted phenyl group (Scheme 10). The Suzuki coupling was again employed; compound **28** underwent the coupling reaction with 2,6-dimethylphenylboronic acid in the presence of a palladium catalyst [Pd(PPh_3_)_4_] and potassium carbonate (K_2_CO_3_) to furnish **30** in 60% yield.

In the ^1^H NMR spectrum of **30**, the singlets for the two methyl groups appear at very different chemical shifts: 2.29 and 1.12 ppm. The difference in chemical shift between the two singlets is 1.17 ppm. On the basis of calculations described below, we conclude that the higher field resonance (1.12 ppm) corresponds to the methyl group over the concave side, whereas the lower field resonance (2.29 ppm) corresponds to the methyl group over the convex side.

**Figure 10:**
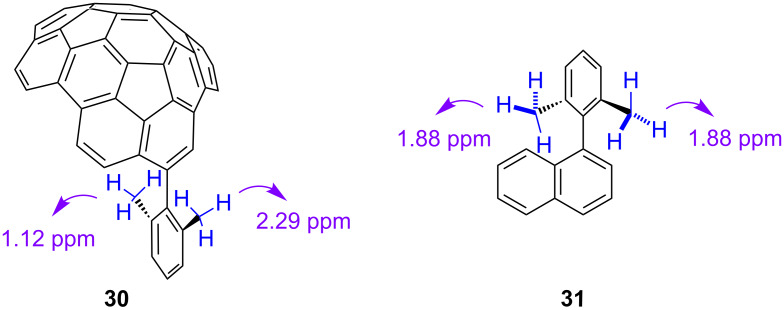
Chemical shifts of *ortho*-methyl groups in **30** and **31**.

Since the walls of circumtrindene consist of three naphthalene units, a 2,6-dimethylphenyl substituted naphthalene **31** was synthesized as a reference compound [61]. The naphthalene units of circumtrindene are arched, but the naphthyl group of **31** is planar; therefore, it provides a good comparison for shielding and de-shielding effects. The ^1^H NMR spectrum of reference compound **31** shows that the methyl groups are magnetically equivalent, as anticipated, giving rise to a single peak at 1.88 ppm (Figure 10). A comparison between **30** and **31** reveals that the signal of the methyl group hanging over the concave side of circumtrindene is shifted upfield by 0.76 ppm, whereas that for the methyl group hanging over the convex side is shifted downfield by 0.41 ppm.

To assign the two NMR signals in the circumtrindene derivative, computational studies of the ^1^H NMR chemical shifts were conducted (GIAO method at the B3LYP/6-31G** level of theory). The results of the calculation show good agreement between the experimentally obtained chemical shifts and those calculated, both for **30** and for reference compound **31** (Table 1). The data clearly indicate that the resonance of the methyl group over the concave face is the one shifted upfield by 0.76 ppm and that the signal for the methyl group over the convex face is the one shifted downfield by 0.41 ppm. Thus, the concave face is substantially more shielding than the convex face. These data reveal a striking difference in the magnetic environments over the two different faces – convex and concave – of circumtrindene, which is a dramatic consequence of the curved π-system.

**Table 1:** Calculated (B3LYP/6-31G**) and experimentally obtained NMR chemical shifts of methyl groups in compounds **30** and **31**.

Compound	δ_exp_ (ppm)	δ_calc_ (ppm)

1-(2,6-dimethylphenyl)naphthalene (**31**)	1.88	1.94
1-(2,6-dimethylphenyl)circumtrindene (**30-endo CH_3_**)	1.12	1.13
1-(2,6-dimethylphenyl)circumtrindene (**30-exo CH_3_**)	2.29	2.32

## Conclusion

Bowl-shaped geodesic polyarenes can be considered the missing links between the “classic” flat PAHs and the spheroidal fullerenes. The present study has shown that open geodesic polyarenes can exhibit chemistry inherent to both classes of aromatics. The curved π-system induces unequal electron densities and unique magnetic environments on the two faces of circumtrindene, significant strain energy in the molecule, and non-identical bond lengths. The lowered LUMO energy and the interior localization of the LUMO coefficients enabled site-selective interior functionalization of circumtrindene by fullerene-type chemistry. On the other hand, the edge carbons, which are not present in fullerenes, still possess reactivity of common planar PAHs. Peripheral functionalization was conducted on those edge carbons, which allowed the π-system to be extended to make a larger bowl indenocircumtrindene (**25**), and the magnetic environments of the two different faces were probed. Computational studies not only supported the experimental data but also provided deeper insights into this curved polycyclic aromatic fullerene fragment.

## Supporting Information

File 1 Experimental procedures and characterization data for all new compounds.
