# Supplementary material for: Site-selective covalent functionalization at interior carbon atoms and on the rim of circumtrindene, a C36H12 open geodesic polyarene
Source: Beilstein J Org Chem. 2014 Apr 28;10:956–68. doi: 10.3762/bjoc.10.94 (PMC4077463; doi:10.3762/bjoc.10.94)
Supplement: File 1 — Experimental procedures and characterization data for all new compounds. [file Beilstein_J_Org_Chem-10-956-s001.pdf]

Supporting Information

for

Site-selective covalent functionalization at interior carbon atoms and on the rim of circumtrindene, a $C_{36}H_{12}$ open geodesic polyarene

Hee Yeon Cho, Ronald B. M. Ansems and Lawrence T. Scott*

Address: Department of Chemistry, Merkert Chemistry Center, Boston College, Chestnut Hill, Massachusetts 02467-3860, USA

Email: Lawrence T. Scott* - lawrence.scott@bc.edu

*Corresponding author

Experimental procedures and characterization data for all new compounds

General Information

All reactions were performed in oven- or flame-dried glassware fitted with rubber septa under a positive pressure of nitrogen, unless otherwise stated. Air- and moisture-sensitive liquids were transferred via syringe or stainless steel cannula. Organic solvents were concentrated by rotary evaporation at various temperatures, unless otherwise noted. All work-up and purification procedures were carried out with reagent grade solvents under typical bench-top conditions. Analytical thin-layer chromatography (TLC) was performed using glass plates, pre-coated with silica gel 60 F_{254} (0.25 mm thickness) impregnated with a fluorescent indicator (254 nm). TLC plates were visualized by exposure to UV (ultraviolet) light, and then were stained with phosphomolybdic acid (PMA) in ethanol, potassium permanganate ($KMnO_4$) in water, or cerium(IV) sulfate and ammonium molybdate in sulfuric acid (CAM). Liquid chromatography was performed using forced flow (flash chromatography) [1] on silica gel (porosity = 60 Å, particle size = 32–63 µm) purchased from Sorbent Technologies. Medium pressure gradient chromatography was performed on a Teledyne Isco CombiFlash automated flash chromatography system with a 200–780 nm UV–vis variable wavelength detector.

All commercially available chemicals and solvents were purchased from Sigma Aldrich, Acros, Strem, Alfa Aesar, Fisher, or TCI America and were used without purification with the following exceptions: Tetrahydrofuran (THF), dichloromethane (CH_2Cl_2), toluene, *N*,*N*-dimethylacetamide (DMAc), 1,2-dichlorobenzene (*o*-DCB), and carbon disulfide (CS_2) were dried and purified using a solvent purification system from Innovative Technology Inc.

Proton nuclear magnetic resonance (^{1}H NMR) spectra were recorded on either a Varian INOVA 500 (500 MHz) or a Varian VNMR 500 (500 MHz) at 23 °C unless specified otherwise. Proton chemical shifts are reported in ppm (parts per million, δ scale) downfield from tetramethylsilane and are referenced to residual protium in the NMR solvent as the internal standard ($CHCl_3$: 7.26 ppm, C_6H_6: 7.16 ppm). Data are reported as follows: chemical shift, integration, multiplicity (s = singlet, d = doublet, t = triplet, q = quartet, qn = quintet, br = broad, and m = multiplet), coupling constant (*J*) in Hertz (Hz), and assignment. Carbon nuclear magnetic resonance (^{13}C NMR) spectra were recorded on either a Varian INOVA 500 (125 MHz) or a Varian VNMR 500 (125 MHz) with complete proton decoupling at 23 °C unless otherwise stated. Carbon chemical shifts are reported in ppm (parts per million, δ scale) downfield from tetramethylsilane and are referenced to the NMR solvent resonance as the internal standard ($CDCl_3$: 77.16 ppm, C_6D_6: 128.06 ppm).

Melting points were determined with a Thomas–Hoover Unimelt capillary melting point apparatus and are uncorrected. Infrared (IR) spectra were recorded on a Bruker FT-IR Alpha (ATR Mode) spectrophotometer, υ_{max} cm^{-1}. Data are represented as follows: frequency of absorption (cm^{-1}) and intensity of absorption (s = strong, m = medium, w = weak, and br = broad). Low-resolution mass spectrometric analyses were performed using a Thermo Electron Corporation Finnigan Trace GC Ultra gas chromatograph unit connected to a Thermo Electron Corporation Finnigan Trace DSQ mass spectrometer with direct inlet capabilities. High-resolution mass spectra (HRMS) were obtained by the Boston College Mass Spectrometry Center using various TOF instruments.

1-(7-Chloronaphthalen-1-yl)ethan-1-one (14)

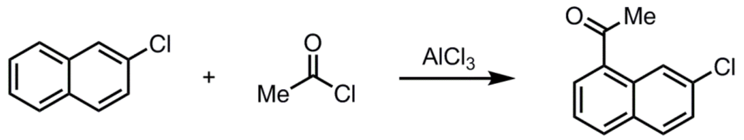

To an oven-dried round-bottomed flask, 2-chloronaphthalene (**13**, 7.2 g, 44 mmol) was dissolved in dichloromethane (73 mL, 0.6 M). The resulting solution was cooled to −10 °C, and then aluminum chloride (18 g, 133 mmol) was added to the solution. The resulting mixture was then cooled to −78 °C. At this temperature, acetyl chloride (7.1 g, 90 mmol) was added, and the reaction mixture was stirred at the same temperature for 10 h. The reaction was quenched with a dilute HCl solution, and the resulting solution was extracted with dichloromethane. The solvent was removed in vacuo to afford the title compound. The crude material was purified by column chromatography on silica gel (90% yield, 8.0 g, white solid). ^{1}H NMR (500 MHz, $CDCl_3$): δ 8.88 (d, *J* = 2.0 Hz, 1H), 8.01 (d, *J* = 7.5 Hz, 1H), 7.89 (d, *J* = 8.0 Hz, 1H), 7.80 (d, *J* = 8.5 Hz, 1H), 7.51 (t, *J* = 8.0 Hz, 1H), 7.49 (d, *J* = 7.5 Hz, 1H), 2.75 (s, 3H). ^{13}C NMR (125 MHz, $CDCl_3$): δ 201.1, 134.5, 134.1, 133.1, 132.2, 130.8, 130.0, 129.8, 127.5, 125.4, 124.6, 29.8. The characterization data were in agreement with literature values [2].

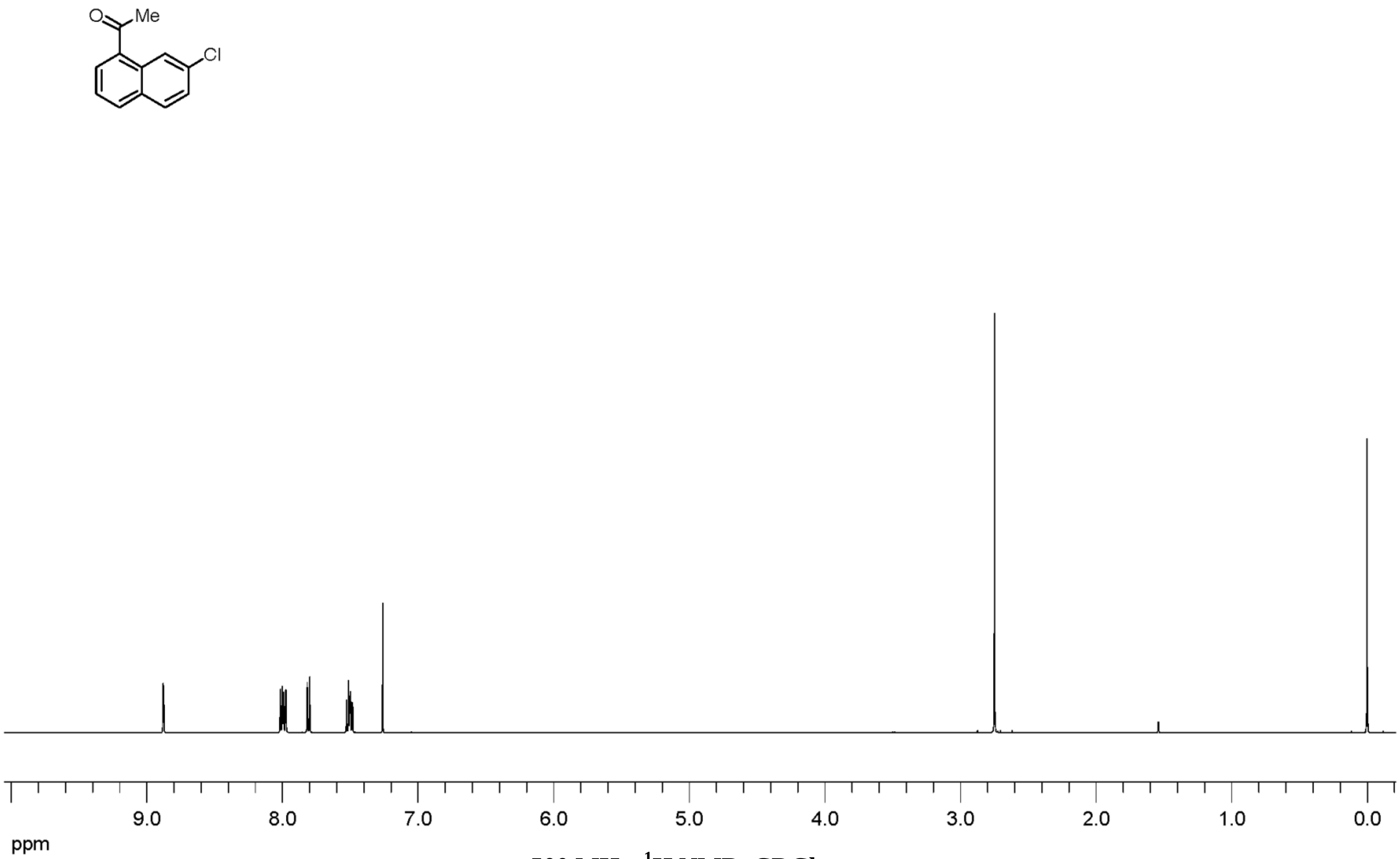

500 MHz, ^{1}H NMR, $CDCl_3$

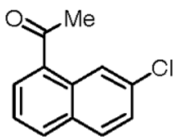

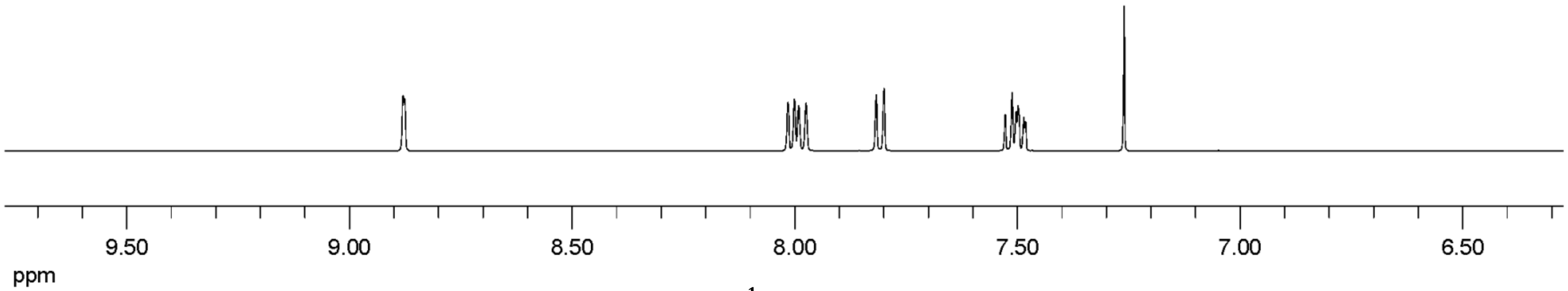

500 MHz, ^{1}H NMR, $CDCl_3$

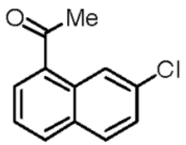

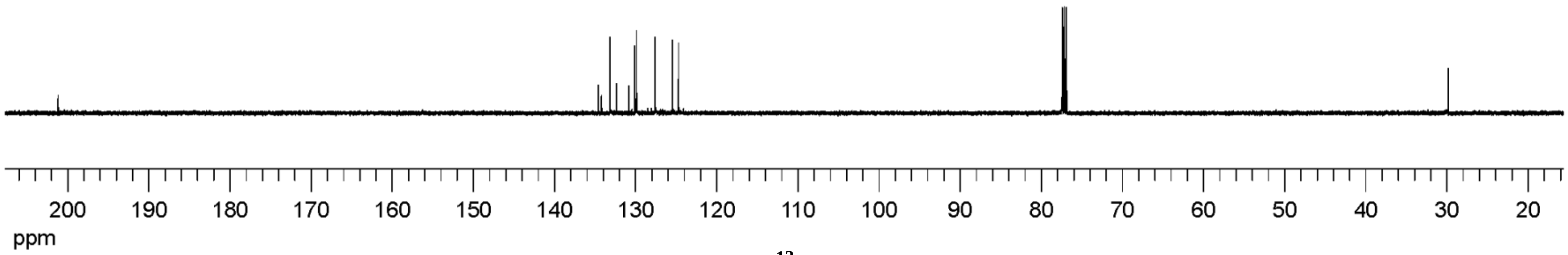

125 MHz, ^{13}C NMR,

Methyl 2-(7-chloronaphthalen-1-yl)acetate (15)

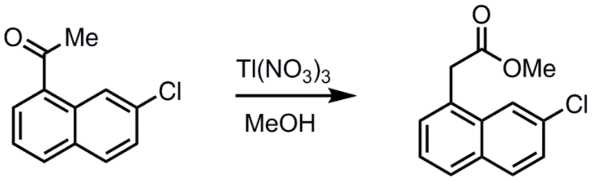

In a flame-dried round-bottomed flask were placed 2.4 g (5.3 mmol) of thallium(III) nitrate (TTN), methanol (21 mL, 0.25 M), perchloric acid (11 mL, 0.5 M), and dichloromethane (11 mL, 0.5 M). Then, 1.1 g (5.3 mmol) of the starting material, 1-(7-chloronaphthalen-1-yl)ethan-1-one (**14**), was slowly added to the reaction mixture. The mixture was then stirred at ambient temperature for 10 h. After this time, the resulting thallium(I) was removed by vacuum filtration, and the filtered reaction mixture was diluted in water. The organic layer of the mixture was separated from the aqueous layer, and the remaining aqueous layer was extracted with dichloromethane. The combined organic layers were washed with brine and dried with anhydrous Na_2SO_4. The drying agent was removed by filtration, and the solvent was evaporated in vacuo. The crude material was purified by column chromatography on silica gel (hexanes/EtOAc) to afford methyl 2-(7-chloronaphthalen-1-yl)acetate (**15**) as a dark yellow oil in quantitative yield (1.2 g). ^{1}H NMR (500 MHz, $CDCl_3$): δ 7.96 (d, J = 1.5 Hz, 1H), 7.80 (d, J = 8.5 Hz, 1H), 7.78 (m, 1H), 7.45–7.43 (m, 3H), 4.04 (s, 2H), 3.72 (s, 3H). The characterization data were in agreement with literature values [2].

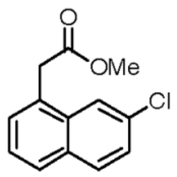

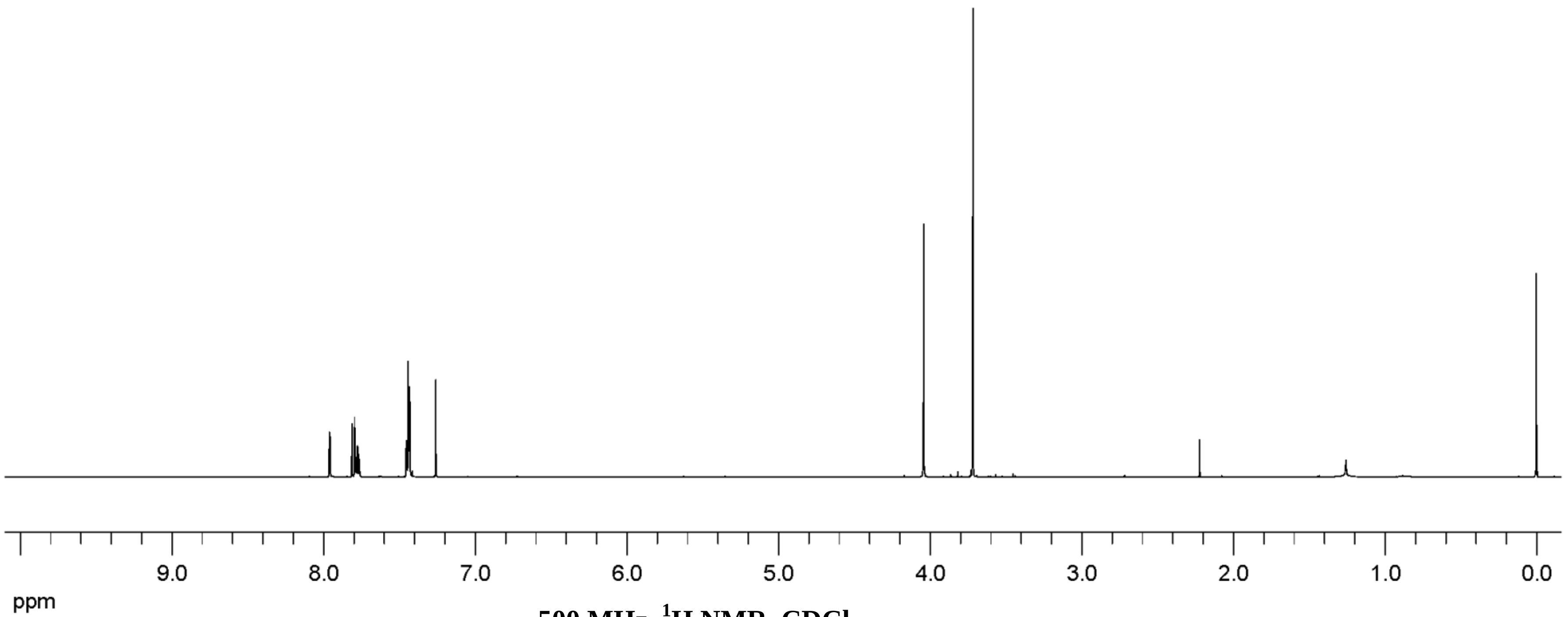

500 MHz, ^{1}H NMR, $CDCl_3$

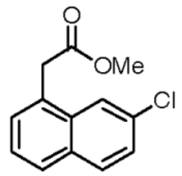

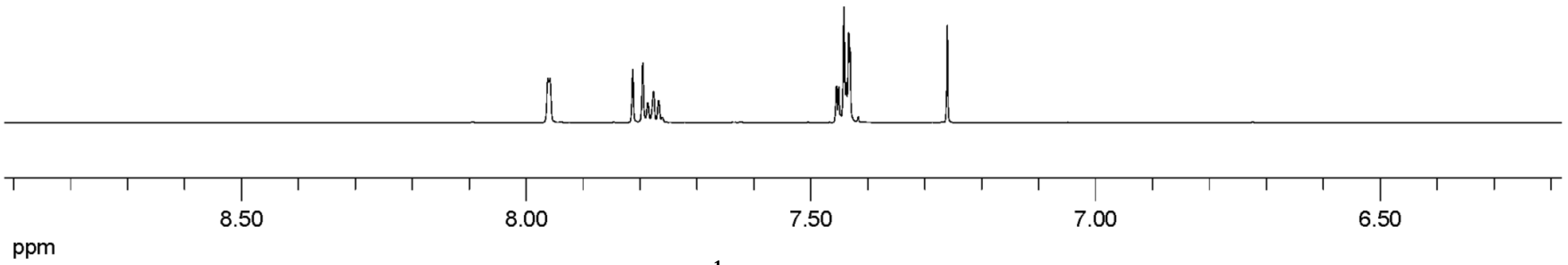

500 MHz, ^{1}H NMR, $CDCl_3$

2-(7-Chloronaphthalen-1-yl)acetic acid (16)

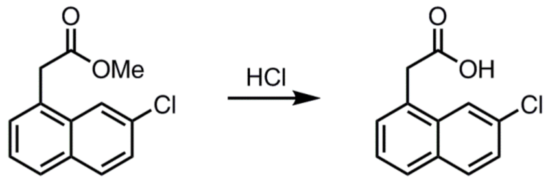

In a round-bottomed flask, 1.2 g (5.0 mmol) of methyl 2-(7-chloronaphthalen-1-yl)acetate (**15**) was dissolved in 5 M HCl (93 mL, 0.054 M) and acetone (93 mL, 0.054 M). A reflux condenser was placed on the flask, and the mixture was heated to reflux for 5 h. After this time, the reaction mixture was cooled to room temperature, and the organic solvents (acetone and methanol) were evaporated in vacuo. The aqueous residue was extracted with dichloromethane, and the solvent was removed to give the crude product. The crude material was dissolved in a dilute NaOH solution, and the solution was washed with benzene. Upon slow addition of 5 M HCl to this basic solution with cooling, the title compound precipitated out of the solution. The product, 2-(7-chloronaphthalen-1-yl)acetic acid (**16**), was obtained by vacuum filtration as an off-white solid in 98% yield (1.1 g). ^{1}H NMR (500 MHz, $CDCl_3$): δ 7.94 (d, *J* = 1.5 Hz, 1H), 7.80 (d, *J* = 9.0 Hz, 1H), 7.78–7.76 (m, 1H), 7.45–7.42 (m, 3H), 4.04 (s, 2H). The characterization data were in agreement with literature values [2].

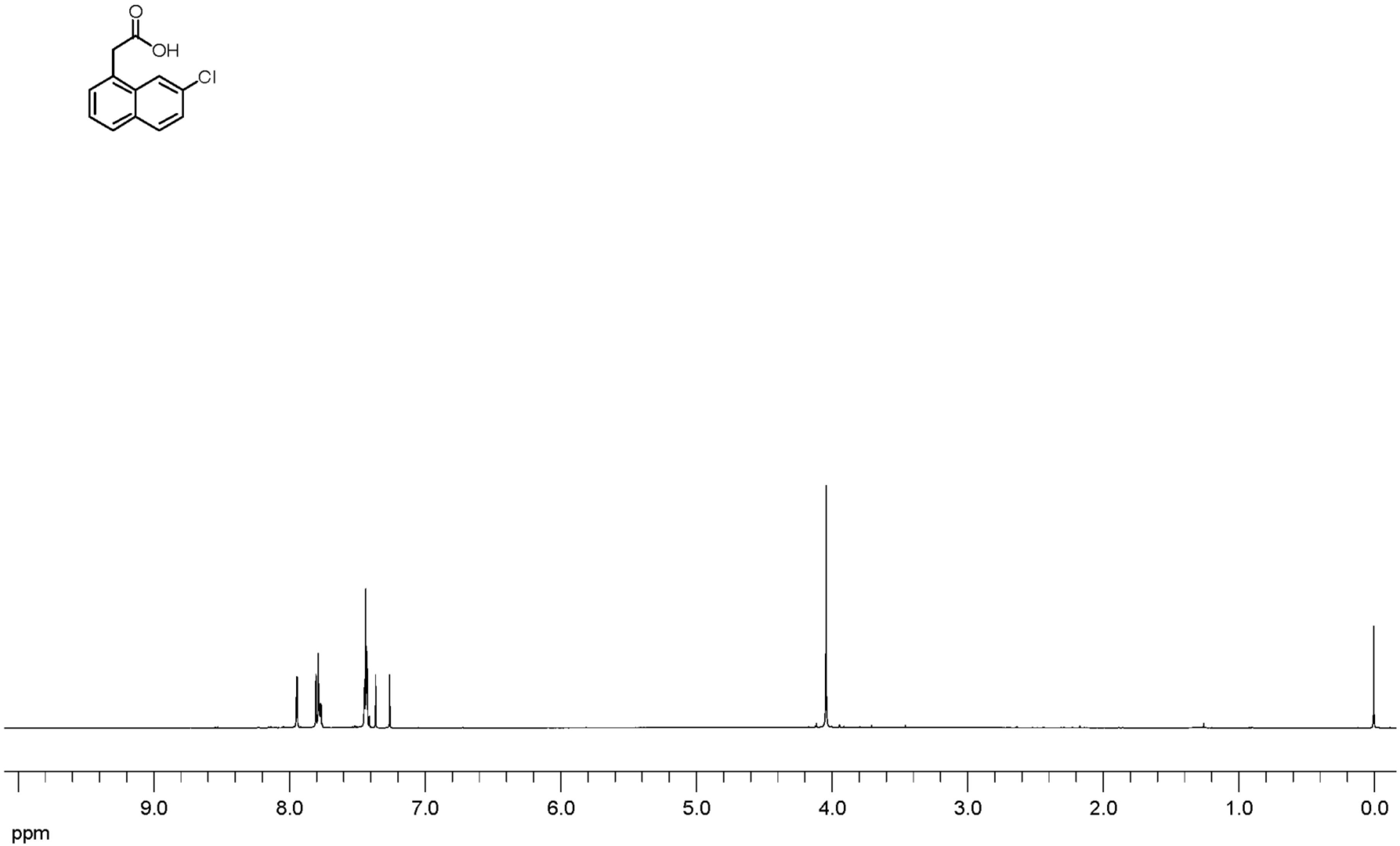

500 MHz, ^{1}H NMR, $CDCl_3$

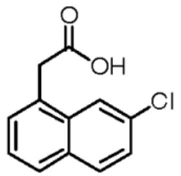

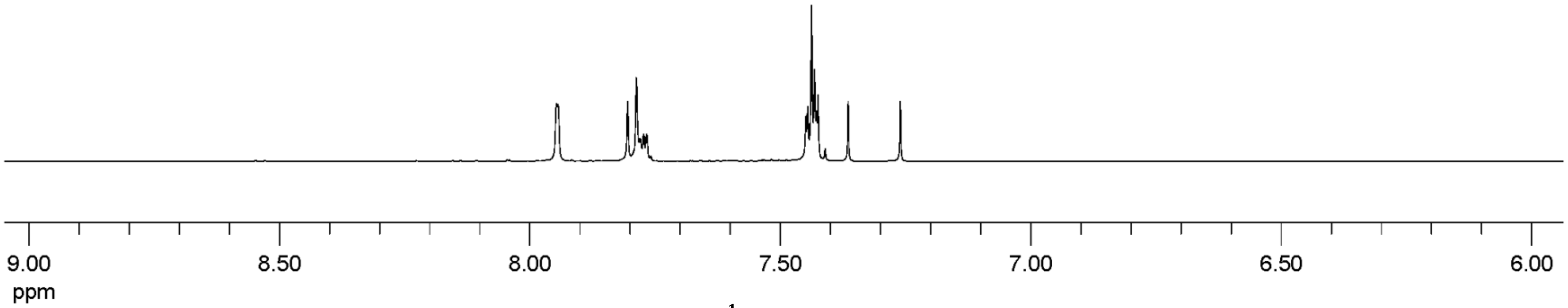

500 MHz, ^{1}H NMR, $CDCl_3$

2-(7-Chloronaphthalen-1-yl)acetyl chloride

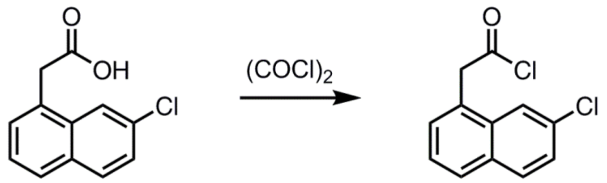

To an oven-dried round-bottomed flask, 7.7 g (35 mmol) of 2-(7-chloronaphthalen-1-yl)acetic acid (**16**) was dissolved in 42 mL (0.8 M) of benzene. To this solution, 14.5 mL (173 mmol) of oxalyl chloride was added, and the reaction mixture was heated at reflux for 5 h. After this time, the volatiles (the solvent and oxalyl chloride) were removed under reduced pressure to afford 2-(7-chloronaphthalen-1-yl)acetyl chloride as a yellow solid. Purification was not performed for this compound; it was directly used as a starting material for the next step. ^{1}H NMR (500 MHz, $CDCl_3$): δ 7.84–7.83 (m, 2H), 7.82 (s, 1H), 7.48–7.46 (m, 3H), 4.53 (s, 2H). ^{13}C NMR (125 MHz, $CDCl_3$): δ 171.6, 133.1, 132.4, 132.1, 130.6, 129.8, 129.1, 127.2, 127.1, 125.8, 122.4, 50.6.

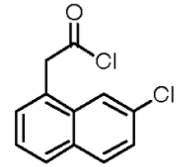

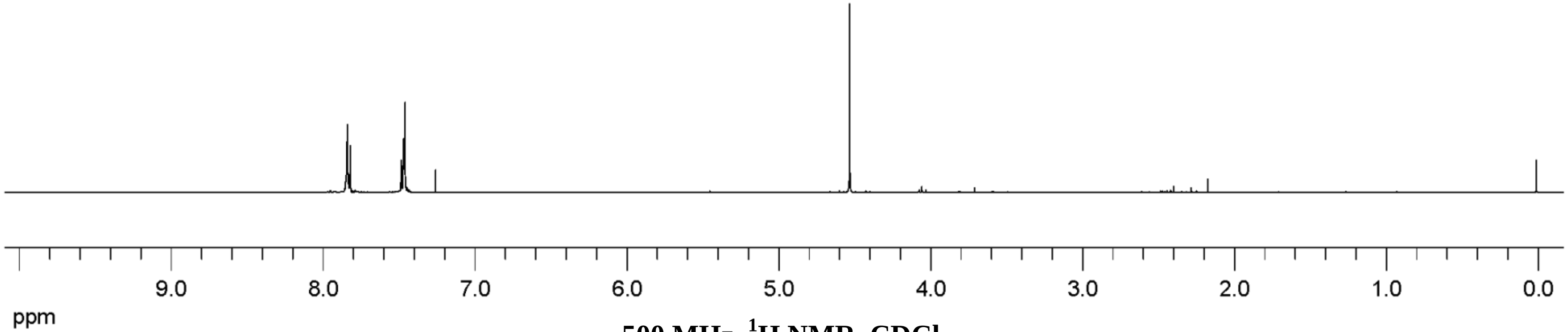

500 MHz, ^{1}H NMR, $CDCl_3$

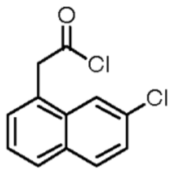

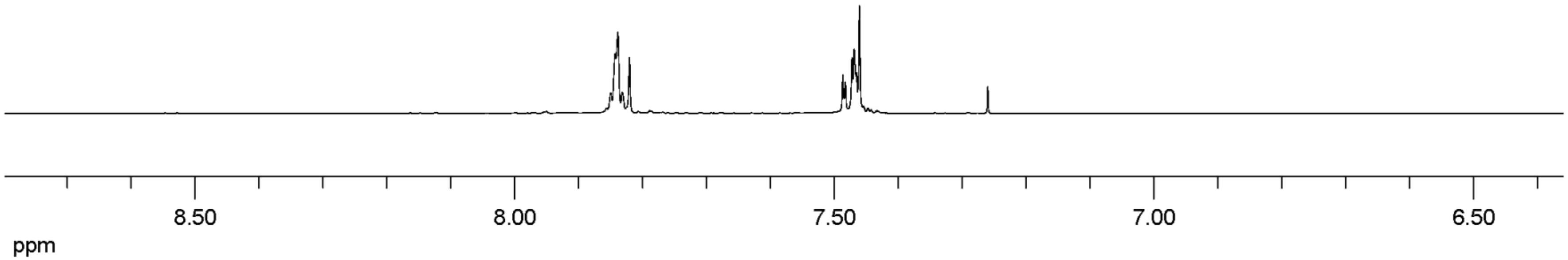

500 MHz, ^{1}H NMR, $CDCl_3$

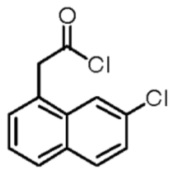

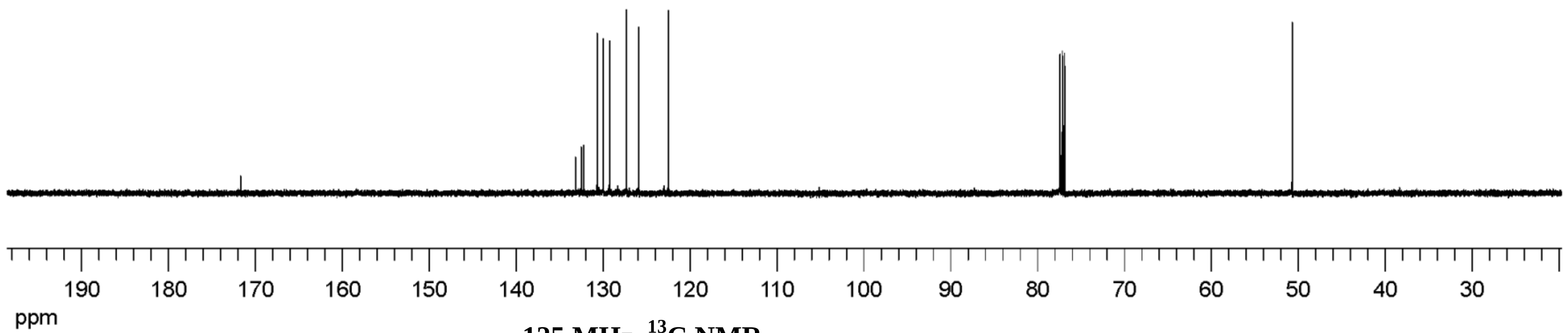

125 MHz, ^{13}C NMR,

8-Chloroacenaphthylen-1(2*H*)-one (17)

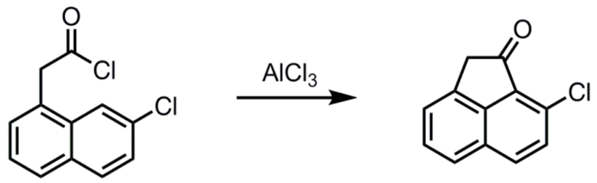

In a flame-dried round-bottomed flask, 2-(7-chloronaphthalen-1-yl)acetyl chloride (7.1 g, 35 mmol) was dissolved in dichloromethane (350 mL, 0.1 M). The resulting solution was cooled to 0 °C in an ice bath, and then aluminum chloride (9.3 g, 70 mmol) was added to the mixture. After the reaction mixture had been stirred at 0 °C for 6 h, it was warmed to room temperature. Then, the mixture was stirred at ambient temperature for 12 h. The reaction was quenched with a dilute HCl solution, and the resulting solution was extracted with dichloromethane. The solvent was removed in vacuo to afford the crude product. The crude material was purified by column chromatography on silica gel to give 8-chloroacenaphthylen-1(2*H*)-one (**17**) as an off-white solid (65% yield over two steps, 4.6 g). ^{1}H NMR (500 MHz, $CDCl_3$): δ 7.98 (d, J = 8.5 Hz, 1H), 7.80 (d, J = 8.5 Hz, 1H), 7.60 (d, J = 8.5 Hz, 1H), 7.58 (t, J = 7.5 Hz, 1H), 7.49 (d, J = 7.0 Hz, 1H), 3.84 (s, 2H). ^{13}C NMR (125 MHz, $CDCl_3$): δ 199.8, 143.7, 133.8, 132.6, 130.2, 130.0, 129.4, 129.1, 128.4, 124.0, 121.9, 42.4. The characterization data were in agreement with literature values [2].

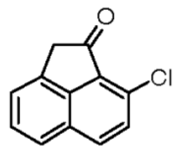

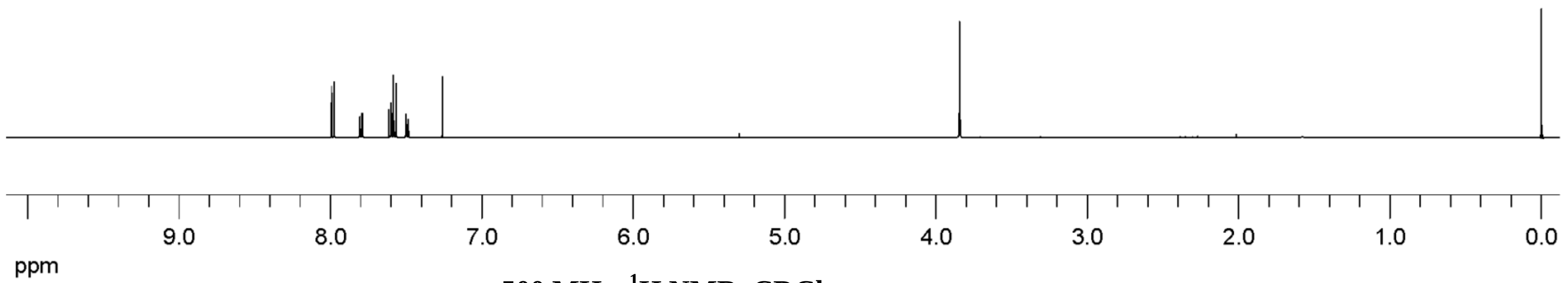

500 MHz, ^{1}H NMR, $CDCl_3$

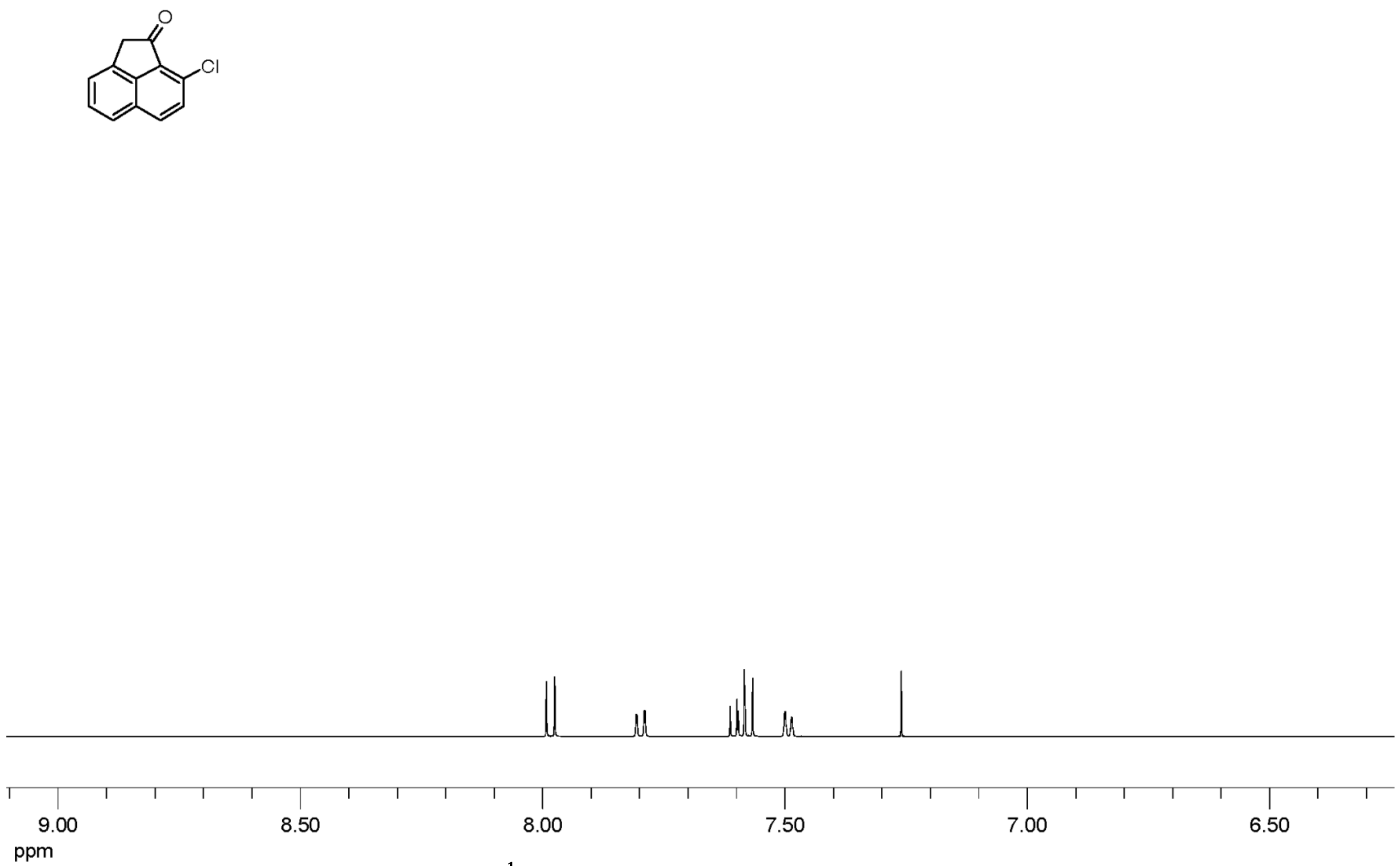

500 MHz, ^{1}H NMR, $CDCl_3$

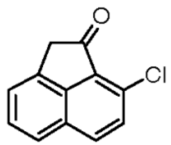

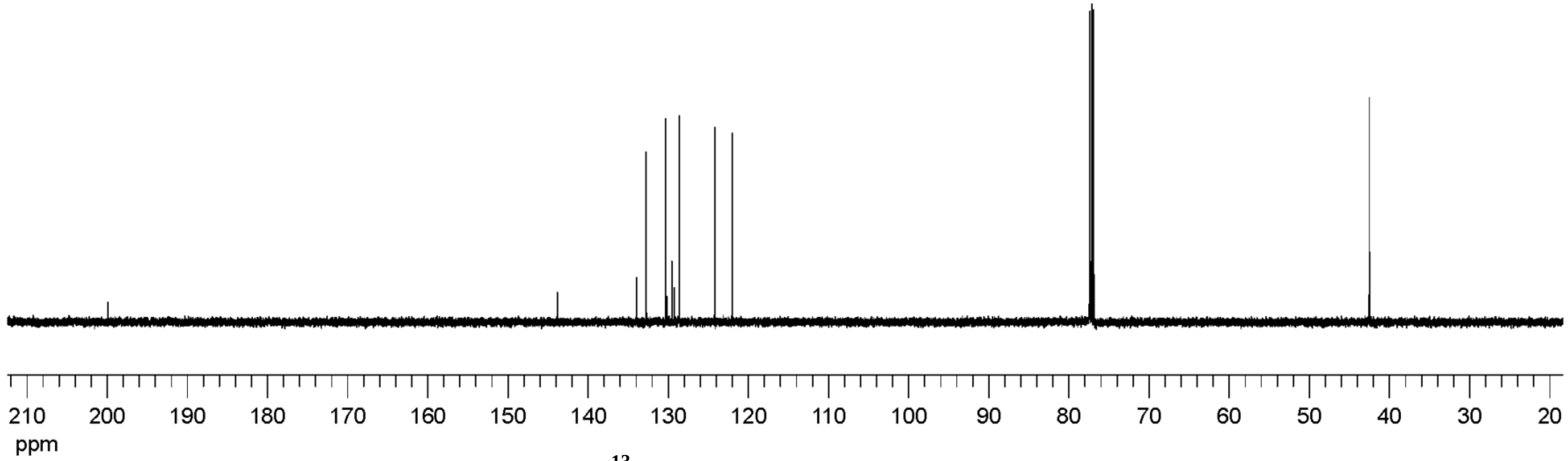

125 MHz, ^{13}C NMR,

1,7,13-Trichlorodecacyclene (12) and circumtrindene (6)

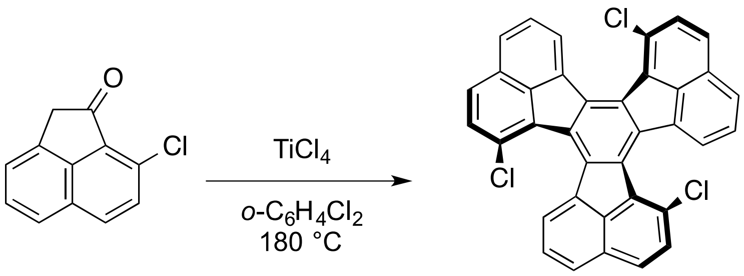

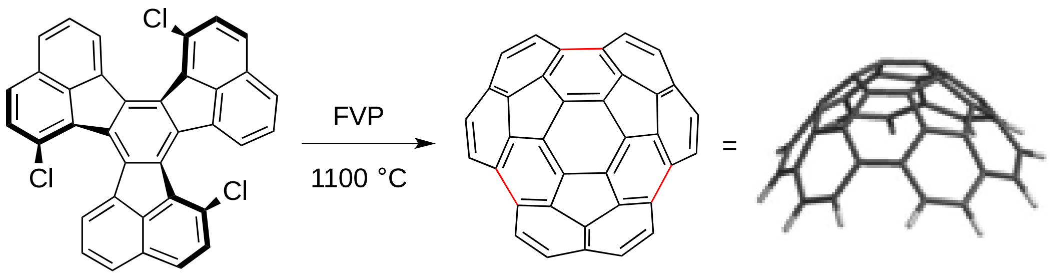

These two steps were carried out as described previously [2]. No improvements were made on either step.

12d,12e-(Bis(ethoxycarbonyl)methylene-bridged)circumtrindene (20)

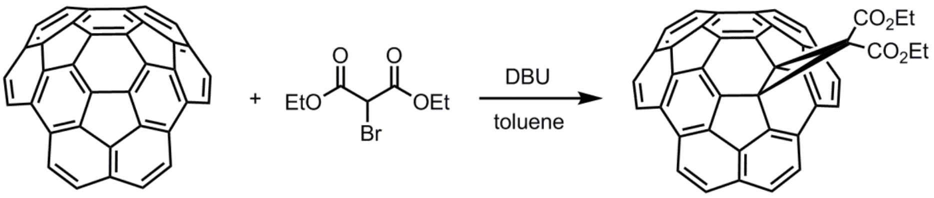

An oven-dried round-bottomed flask, equipped with a magnetic stir bar, was charged with 4.2 mg (0.0095 mmol) of circumtrindene (**6**), 23 mg (0.095 mmol) of ethyl bromomalonate, 14 mg (0.095 mmol) of DBU, and 1.9 mL (0.005 M) of toluene. The reaction mixture was stirred at ambient temperature for 2 h under an inert atmosphere of nitrogen. After this time, the reaction was quenched with trifluoroacetic acid (72 μL, 0.95 mmol), and the organic solvent was evaporated under reduced pressure. The crude material was purified by column chromatography on silica gel with hexanes/ether as an eluent to afford 12d,12e-(bis(ethoxycarbonyl)methylene-bridged)circumtrindene as a yellow solid in 75% yield (4.3 mg). R_f = 0.26 (1:1 hexane:ether). ^{1}H NMR (500 MHz, $CDCl_3$): δ 7.68 (d, *J* = 9.0 Hz, 2H), 7.66 (d, *J* = 8.5 Hz, 2H), 7.33 (d, *J* = 9.0 Hz, 2H), 7.25 (d, *J* = 9.0 Hz, 2H), 7.15 (d, *J* = 9.0 Hz, 2H), 7.12 (d, *J* = 9.0 Hz, 2H), 4.55 (q, *J* = 7.0 Hz, 2H), 4.39 (q, *J* = 7.0 Hz, 2H), 1.45 (t, *J* = 7.0 Hz, 3H), 1.34 (t, *J* = 7.0 Hz, 3H). HRMS (MALDI) calculated for $C_{43}H_{22}O_4$ $[M]^+$: 602.1513, found: 602.1515.

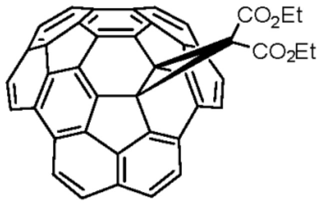

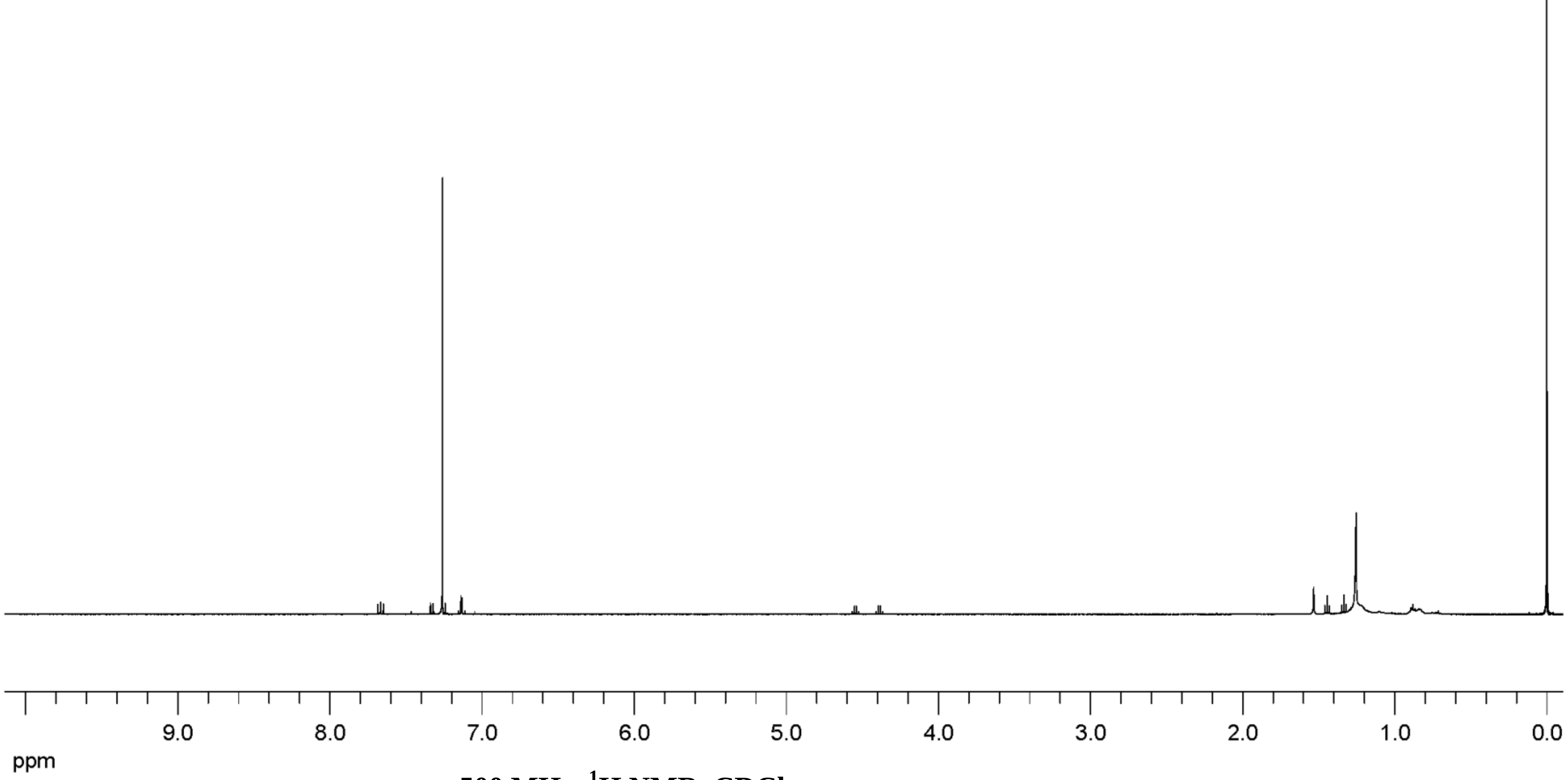

500 MHz, ^{1}H NMR, $CDCl_3$

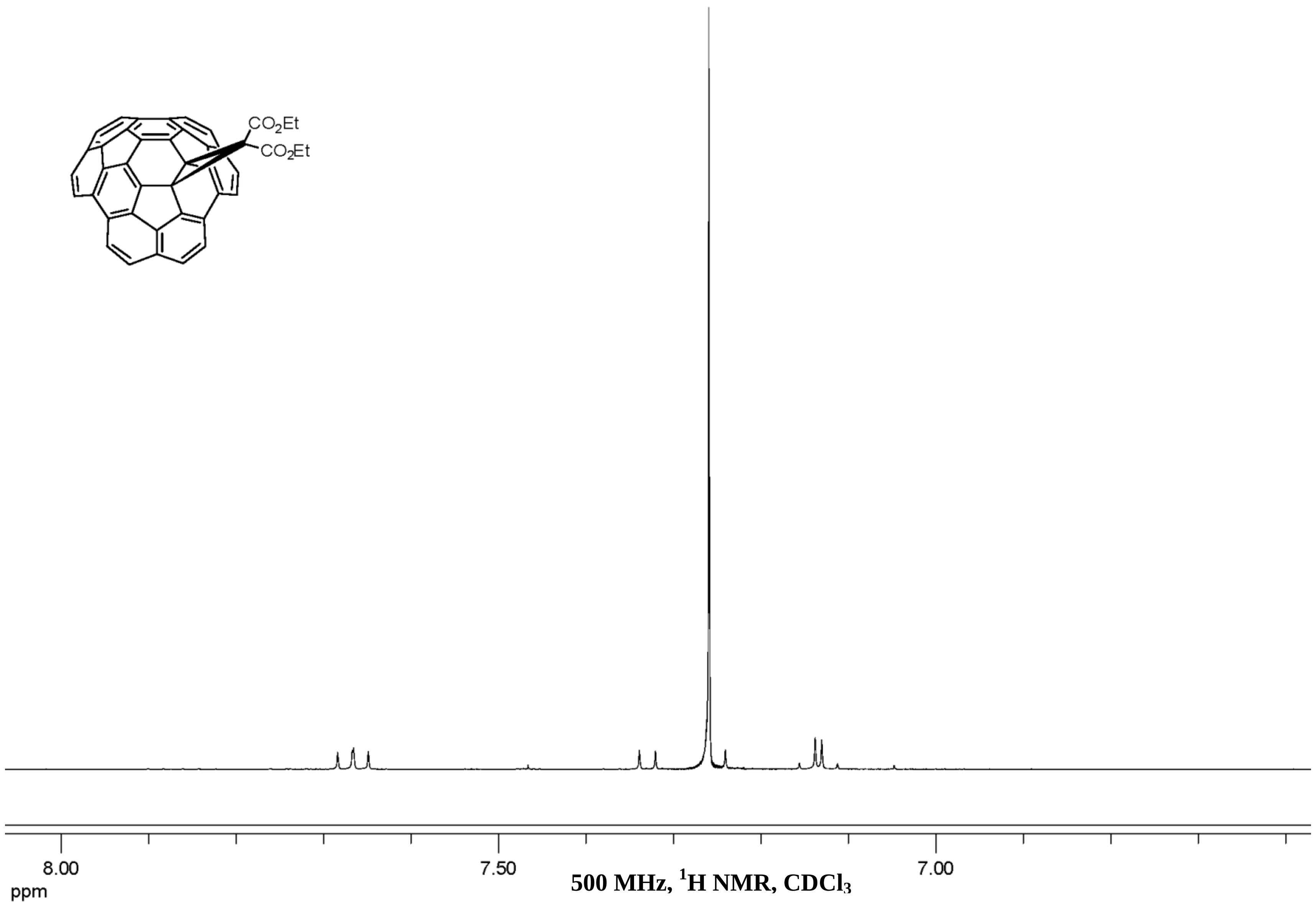

500 MHz, ^1H NMR, $CDCl_3$

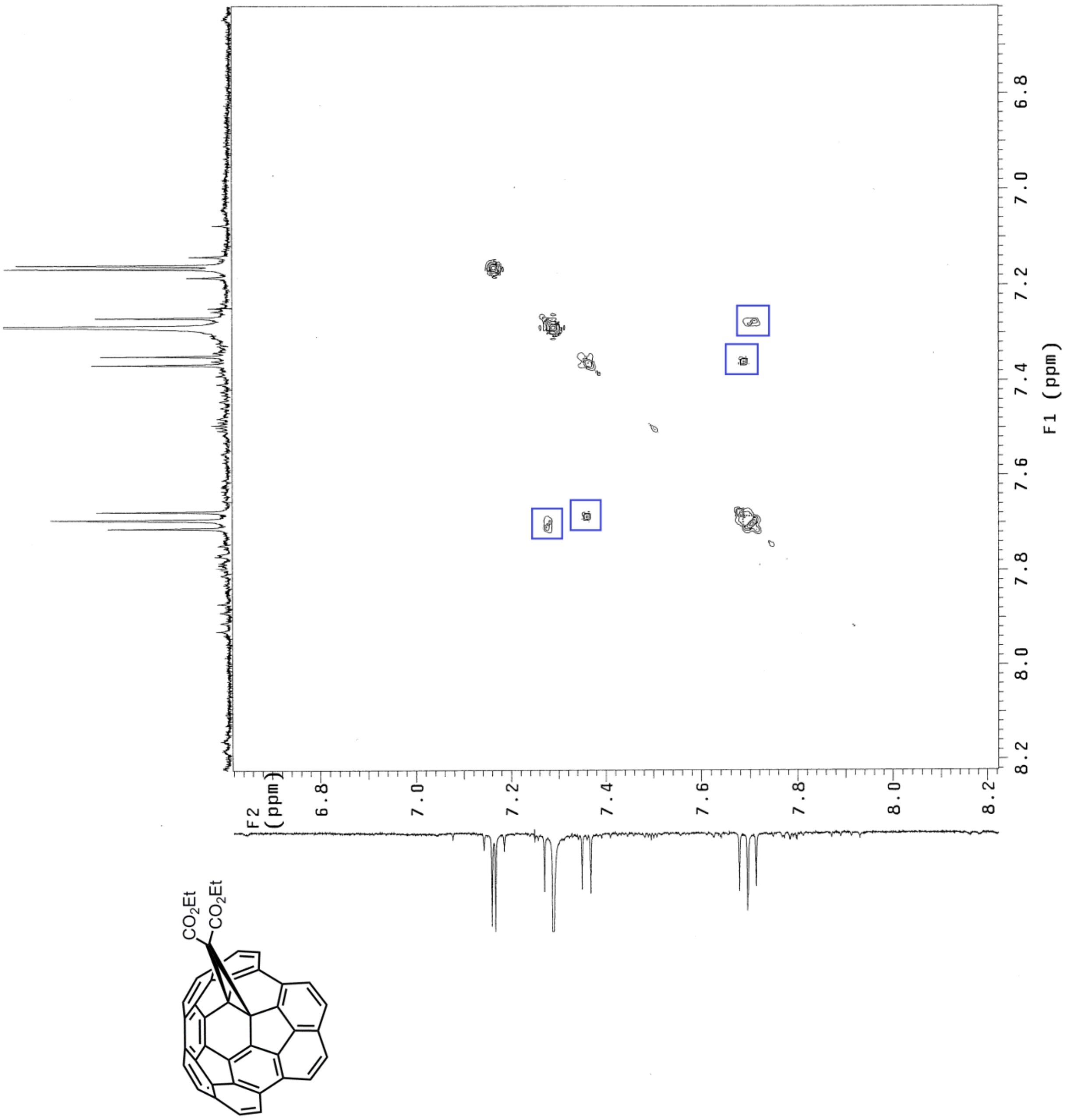
F1 (ppm)
F2 (ppm)
CO2Et
CO2Et

12d,12e-(*N*-methyl-3,4-pyrrolidino)circumtrindene (24)

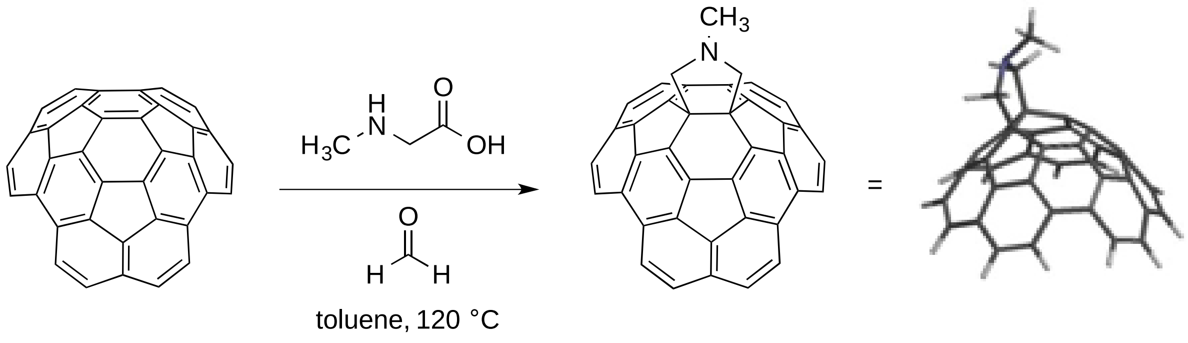

Sarcosine (*N*-methylglycine, 52 mg, 0.58 mmol) and 70 mg (2.33 mmol) of paraformaldehyde were added to a solution of 5.2 mg (0.012 mmol) of circumtrindene (**6**) in 3 mL of toluene. The system was purged with nitrogen, closed from the atmosphere and kept under slight pressure by means of a balloon filled with nitrogen. The reaction mixture was heated to reflux with stirring for 4 h. After cooling to room temperature, the mixture was extracted with dichloromethane, washed with 10% hydrochloric acid, saturated sodium bicarbonate solution and a saturated sodium chloride solution. The organic layer was dried over magnesium sulfate and concentrated to dryness under reduced pressure. Preparative TLC on alumina with 1:1 dichloromethane:hexanes as eluent gave 12d,12e-(*N*-methyl-3,4-pyrrolidino)-circumtrindene (**24**) in 58–66% yield (3.5–4.0 mg) as a yellow solid that slowly decomposed and could not be thoroughly characterized. Attempts to obtain HRMS and ^{13}C NMR spectra were unsuccessful, but the ^{1}H NMR spectrum clearly confirms the structure. ^{1}H NMR (400 MHz, $CDCl_3$): δ 7.68 (d, J = 9.2 Hz, 2H), 7.61 (d, J = 9.2 Hz, 2H), 7.40 (d, J = 8.8 Hz, 2H), 7.18 (d, J = 9.2 Hz, 2H), 7.03 (d, J = 8.8 Hz, 2H), 6.85 (d, J = 8.8 Hz, 2H), 4.24 (d, J = -9.6 Hz, 2H), 4.14 (d, J = -9.6 Hz, 2H), 2.86 (s, 3H).

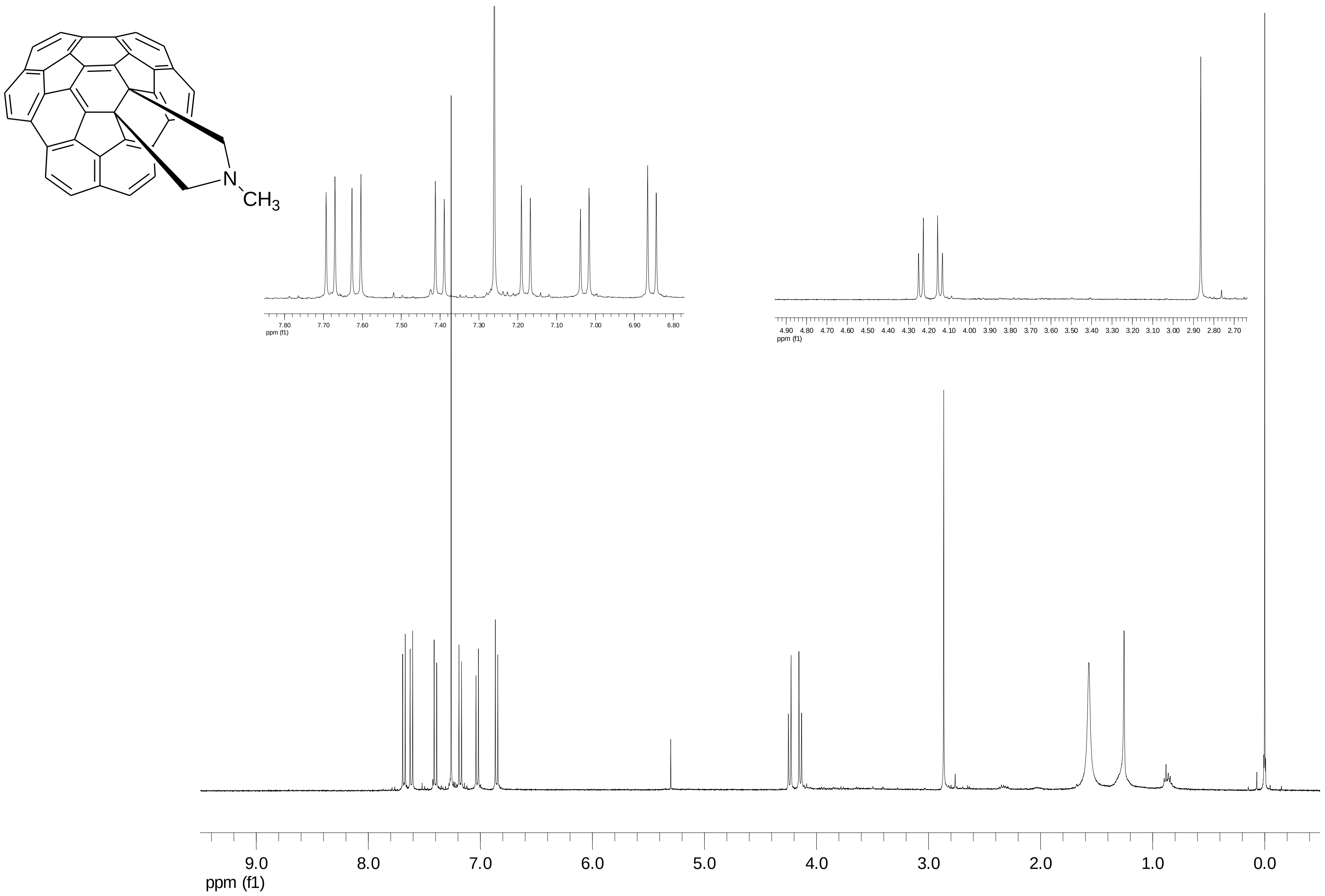
N
CH3
7.80 7.70 7.60 7.50 7.40 7.30 7.20 7.10 7.00 6.90 6.80
ppm (f1)
4.90 4.80 4.70 4.60 4.50 4.40 4.30 4.20 4.10 4.00 3.90 3.80 3.70 3.60 3.50 3.40 3.30 3.20 3.10 3.00 2.90 2.80 2.70
ppm (f1)
9.0 8.0 7.0 6.0 5.0 4.0 3.0 2.0 1.0 0.0
ppm (f1)

1-Bromocircumtrindene (28)

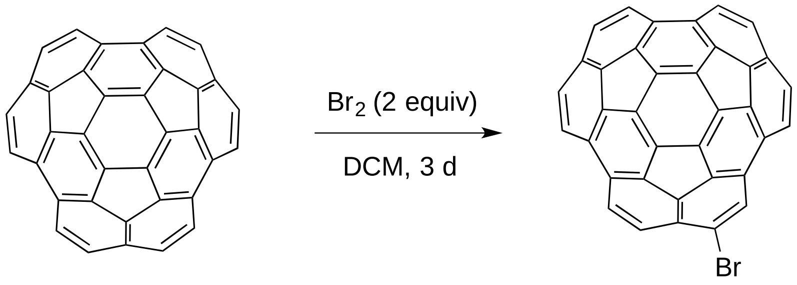

To a small round-bottomed flask with a magnetic stirrer were added 15.6 mg (0.035 mmol) of circumtrindene (**6**) and 7.5 mL of a 1.5 mg/mL (0.070 mmol) solution of bromine in dichloromethane. The flask was sealed with a stopper and Teflon tape to prevent solvent evaporation, and the reaction mixture was stirred for three days at room temperature. Residual bromine was reduced by the addition of a few mL of a 10% aqueous sodium thiosulfate solution. After extraction with dichloromethane, washing with saturated sodium chloride, drying over magnesium sulfate, and evaporation of the solvent under reduced pressure, a yellow solid remained. Preparative TLC (1:4 dichloromethane:hexanes) yielded 18.2 mg (95%) mono-brominated circumtrindene **28** and 1.4 mg (5%) of a mixture of the three dibrominated circumtrindenes. ^{1}H NMR (400 MHz, $CDCl_3$): δ 7.79 (s, 1H), 7.68 (d, J = 9.2 Hz, 1H), 7.61 (d, J = 9.2 Hz, 2H), 7.59 (d, J = 9.2 Hz, 1H), 7.55 (d, J = 8.8 Hz, 1H), 7.40 (d, J = 9.6 Hz, 1H), 7.24 (d, J = 8.8 Hz, 1H), 7.23 (d, J = 8.8 Hz, 1H), 7.21 (d, J = 9.2 Hz, 2H). ^{1}H NMR (400 MHz, CD_2Cl_2): δ 7.84 (s, 1H), 7.72 (d, J = 9.6 Hz, 1H), 7.64 (d, J = 9.2 Hz, 2H), 7.63 (d, J = 9.2 Hz, 1H), 7.57 (d, J = 9.2 Hz, 1H), 7.41 (d, J = 9.2 Hz, 1H), 7.26 (d, J = 9.2 Hz, 1H), 7.26 (d, J = 9.2 Hz, 1H), 7.25 (d, J = 9.2 Hz, 2H). ^{13}C NMR (125 MHz, $CDCl_3$): δ 128.11, 127.47, 127.43, 127.35, 127.24, 126.70, 126.40, 125.85, 125.79, 125.38, 125.32 [Even after 48 h, only the signals for the methine carbons could be seen in the ^{13}C NMR spectrum]. HRMS (EI, 70 eV) calculated for $C_{36}H_{11}Br$ (M^+, ^{79}Br): 522.0044, found: 522.0043.

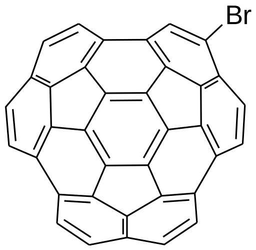
Br

In $CDCl_3$

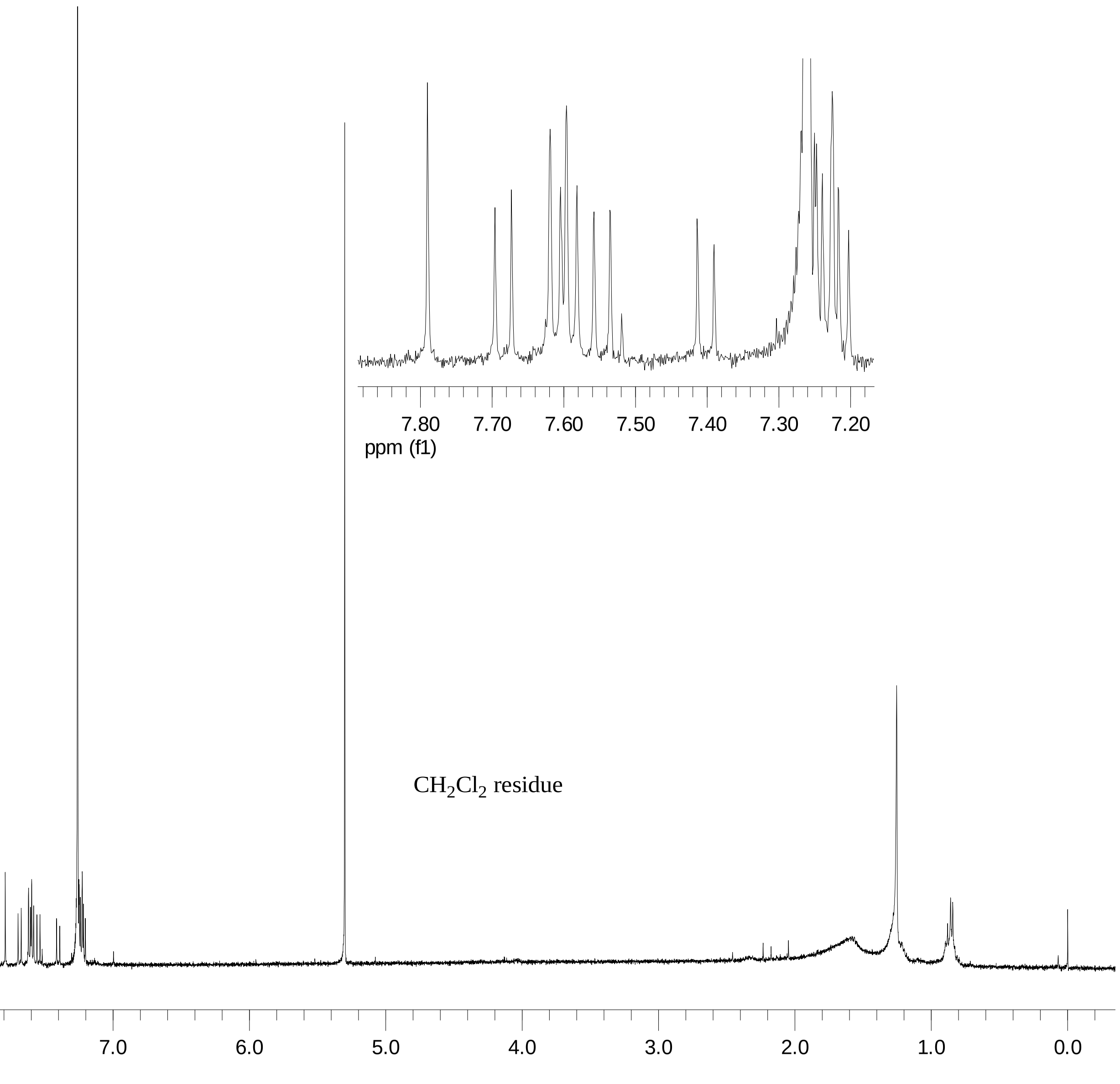
7.80
7.70
7.60
7.50
7.40
7.30
7.20
ppm (f1)
CH2Cl2 residue
9.0
8.0
7.0
6.0
5.0
4.0
3.0
2.0
1.0
0.0
ppm (f1)

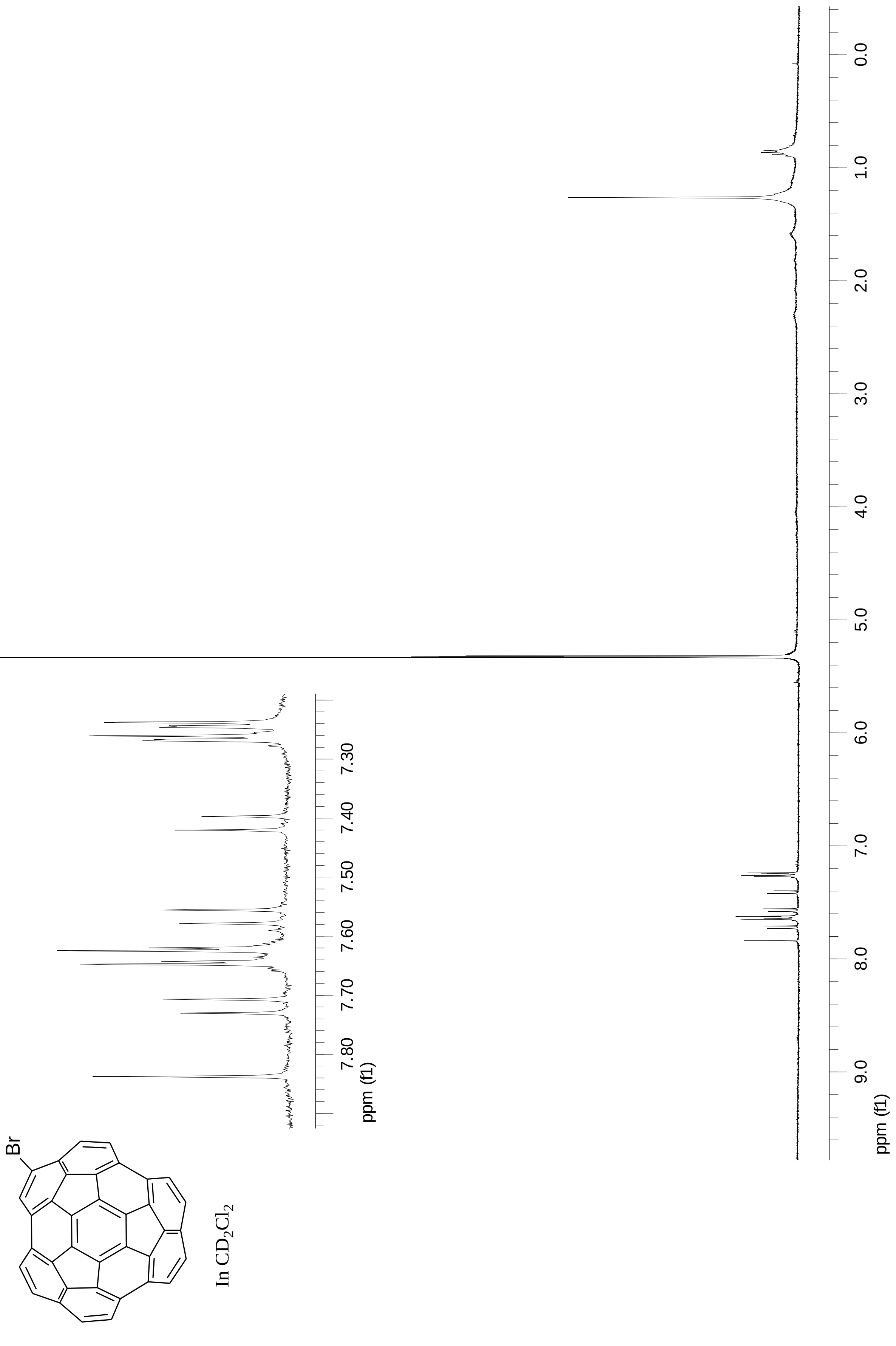
Br
In CD_2Cl_2
7.30
7.40
7.50
7.60
7.70
7.80
ppm (f1)
0.0
1.0
2.0
3.0
4.0
5.0
6.0
7.0
8.0
9.0
ppm (f1)

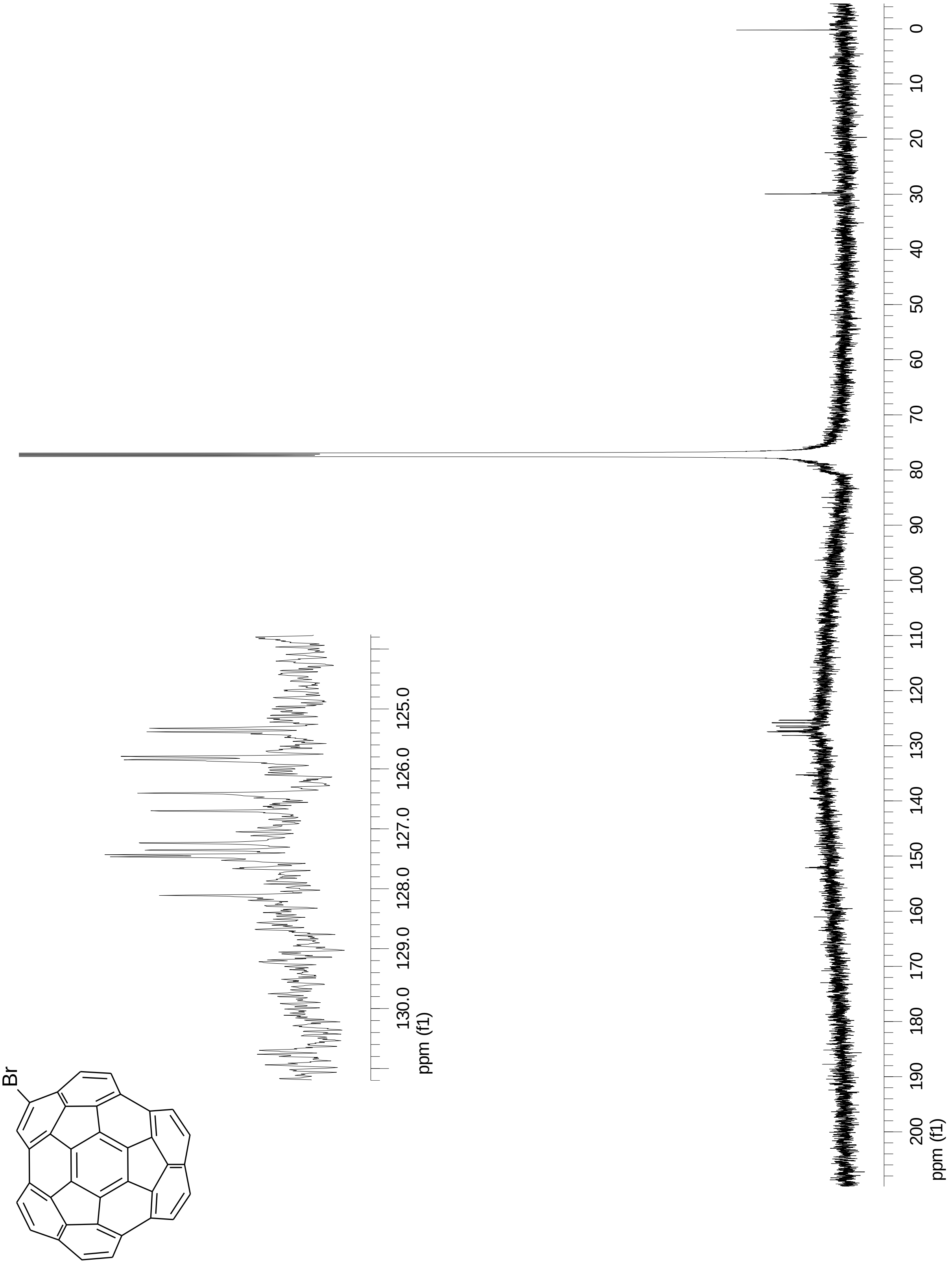

Br
125.0
126.0
127.0
128.0
129.0
130.0
ppm (f1)
0
10
20
30
40
50
60
70
80
90
100
110
120
130
140
150
160
170
180
190
200
ppm (f1)

1-(2-Bromophenyl)circumtrindene (29)

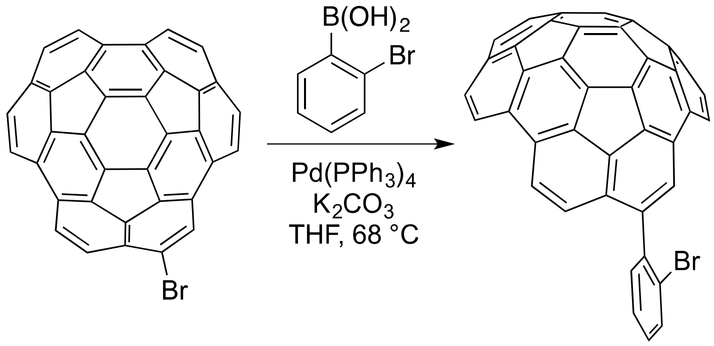

In a 25 mL Schlenk flask equipped with a magnetic stirring bar were mixed approximately 20 mg (0.038 mmol) 1-bromocircumtrindene (**28**), 42 mg (0.21 mmol) 2-bromophenylboronic acid, 7 mg (0.006 mmol) tetrakis(triphenylphosphine)-palladium, 55 mg (0.40 mmol) potassium carbonate, and 2 mL THF. The reaction vessel was purged with nitrogen and lowered into a preheated wax bath. After stirring overnight under reflux, the reaction was quenched with a 10% aqueous hydrochloric acid solution and extracted into dichloromethane. Washing with saturated aqueous sodium bicarbonate and saturated aqueous sodium chloride followed by drying over magnesium sulfate and removal of the solvent under reduced pressure resulted in ca 20 mg (90%) of 1-(2-bromophenyl)circumtrindene (**29**) as a yellow solid. In order to preserve the maximum amount of compound for the pyrolysis step, no further purification was performed. ^{1}H NMR (400 MHz, CD_2Cl_2): δ 7.74 (bd, *J* = 7.6 Hz, 1H), 7.65 (d, *J* = 8.8 Hz, 2H), 7.61 (d, *J* = 8.8 Hz, 1H), 7.59 (d, *J* = 8.8 Hz, 1H), 7.53 (d, *J* = 9.2 Hz, 1H), 7.27 (d, *J* = 9.2 Hz, 1H), 7.26 (d, *J* = 9.2 Hz, 1H), 7.23 (d, *J* = 8.8 Hz, 1H), 7.22 (d, *J* = 8.8 Hz, 1H), 6.83 (d, *J* = 9.2 Hz, 1H), 6.69 (bd, *J* = 7.6 Hz, 1H). HRMS (EI, 70 eV) calculated for $C_{42}H_{15}Br$ (M^{+}, ^{79}Br): 598.0357, found: 598.0351.

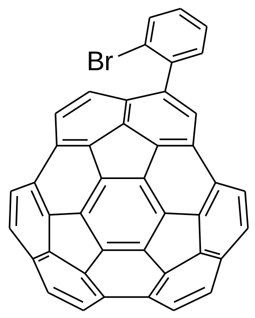

In $CDCl_3$

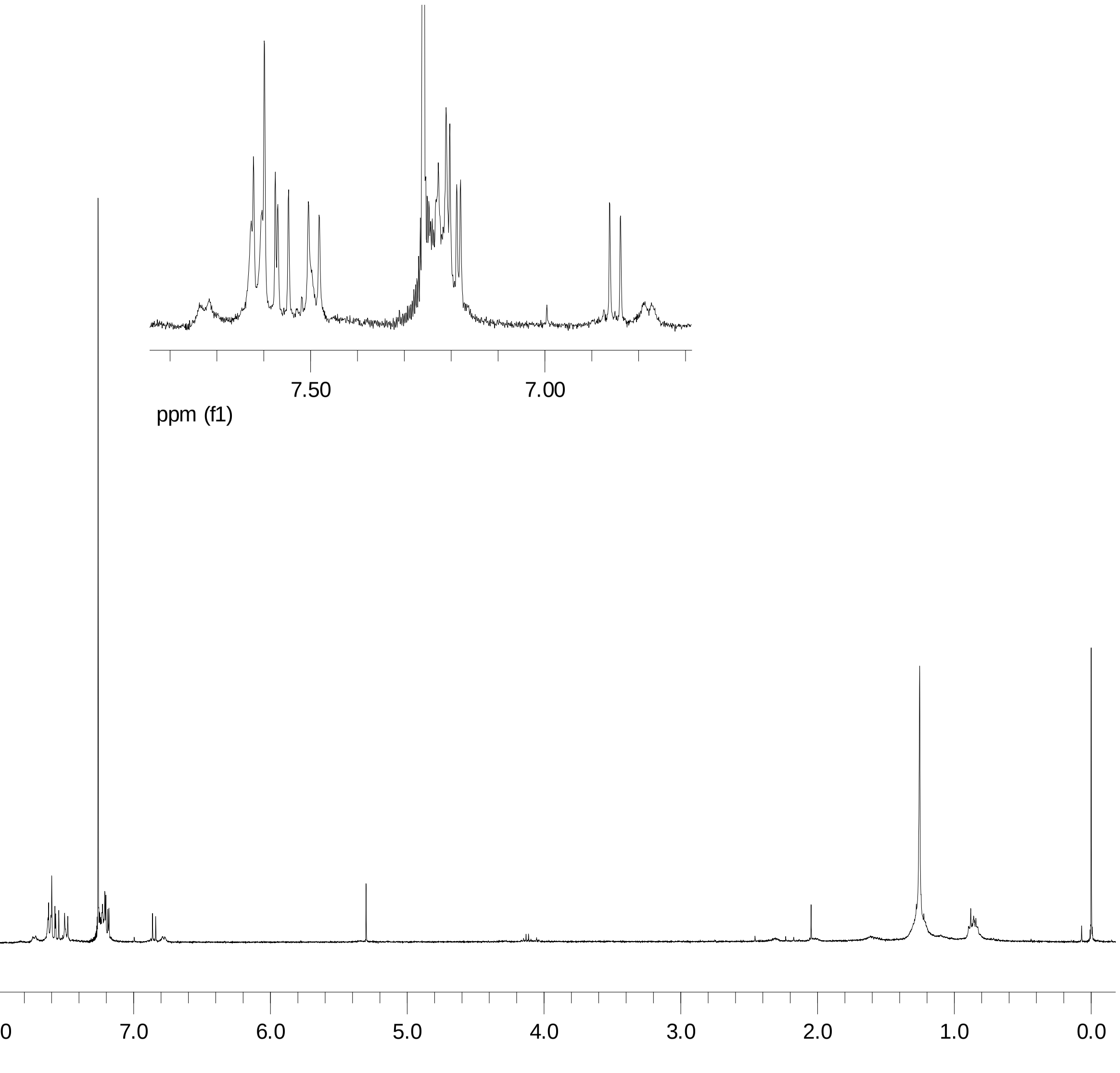

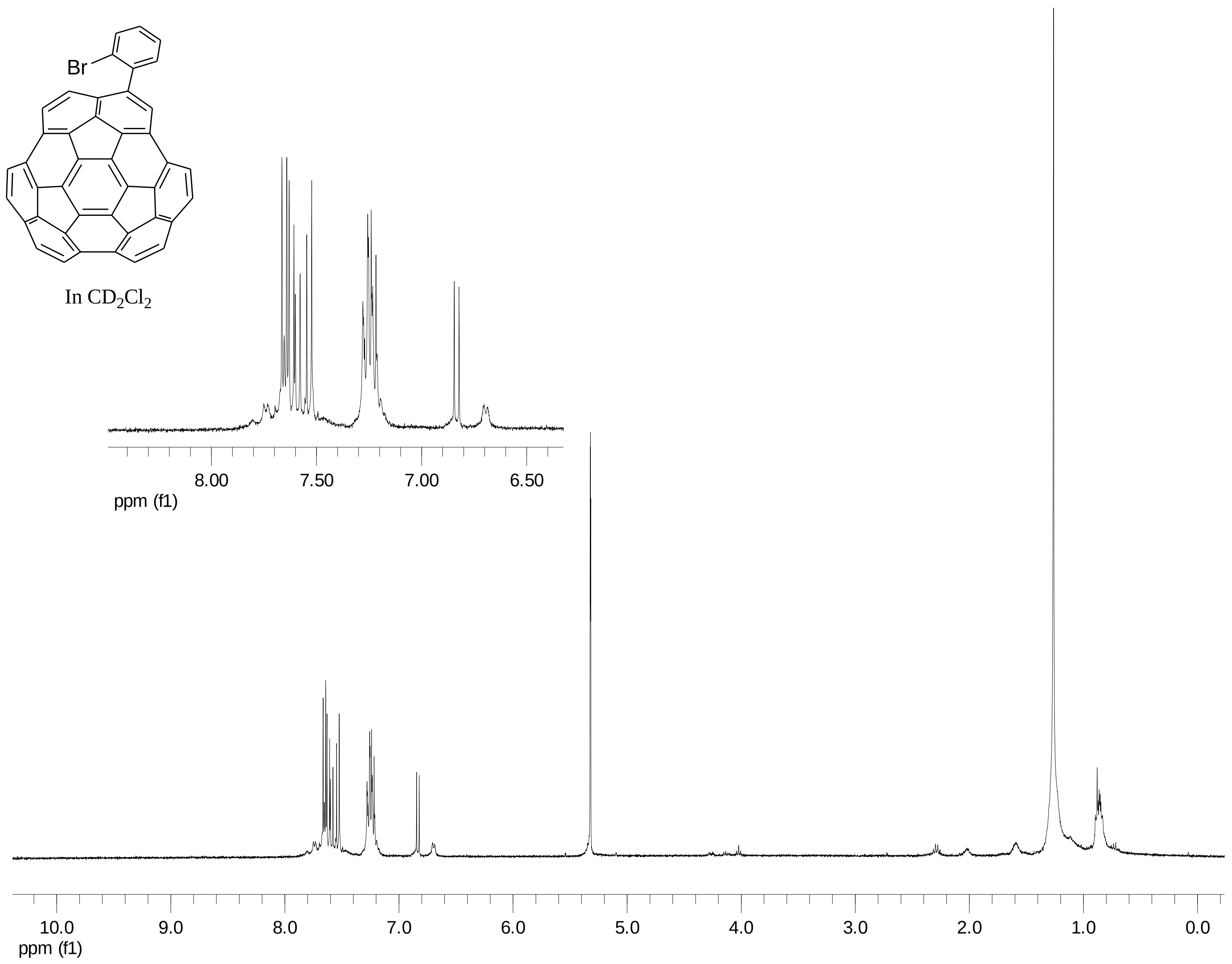

In CD_2Cl_2

Indeno[3,2,1-*f,g*]circumtrindene (25)

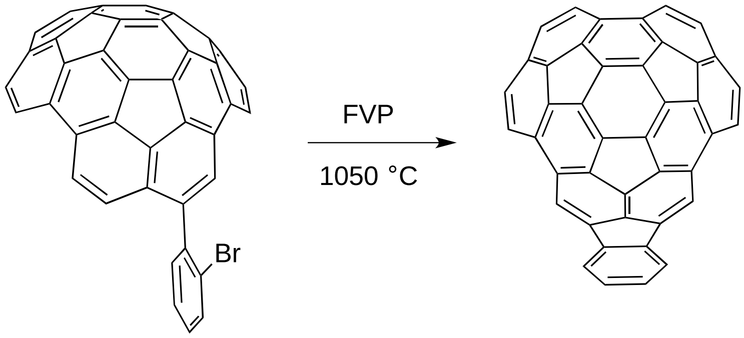

A sample of unpurified 1-(2-bromophenyl)circumtrindene (**29**) (ca 20 mg from the previous reaction) was placed in a quartz sample boat. The boat was placed into the front end of a quartz furnace tube so that it was positioned approximately 2 cm from the entrance of the furnace. The system was closed off from the surrounding environment and placed under high vacuum (1 mmHg) with a nitrogen bleed through a GC capillary on the other end of the system. While heating up the furnace a hairdryer set to cold was aimed at the part of the furnace tube with the sample boat to prevent premature decomposition. As soon as the oven reached the desired temperature of 1050 °C, the hairdryer was removed and replaced with heating tape around the part of the furnace tube that contained the sample boat. The cold traps were filled with liquid nitrogen, and the heating tape was slowly allowed to warm up by adjusting a variable AC controller. Over several hours, the sample sublimed through the tube, and the FVP product was collected on the other side of the oven. Most of the product was circumtrindene (loss of the phenyl group), but a small amount was indeno[3,2,1-*f,g*]circumtrindene (**25**). Repeated purification by preparative TLC (10–20% dichloromethane:hexanes on alumina) failed to completely remove the circumtrindene. Attempts to obtain a HRMS were unsuccessful, because the M^+ signal for **25** was too weak in the sample mixture. ^{1}H NMR (400 MHz, CS_2,

with cyclohexane-d_{12} as lock solvent at 1.38 ppm): δ 6.88 (d, J = 8.8 Hz, 2H), 6.80 (d, J = 8.8 Hz, 2H), 6.72 (s, 2H), 6.66-6.64 (AA´BB´, 2H), 6.44 (d, J = 9.2 Hz, 2H), 6.42 (d, J = 8.8 Hz, 2H), 6.33-6.31 (AA´BB´, 2H).

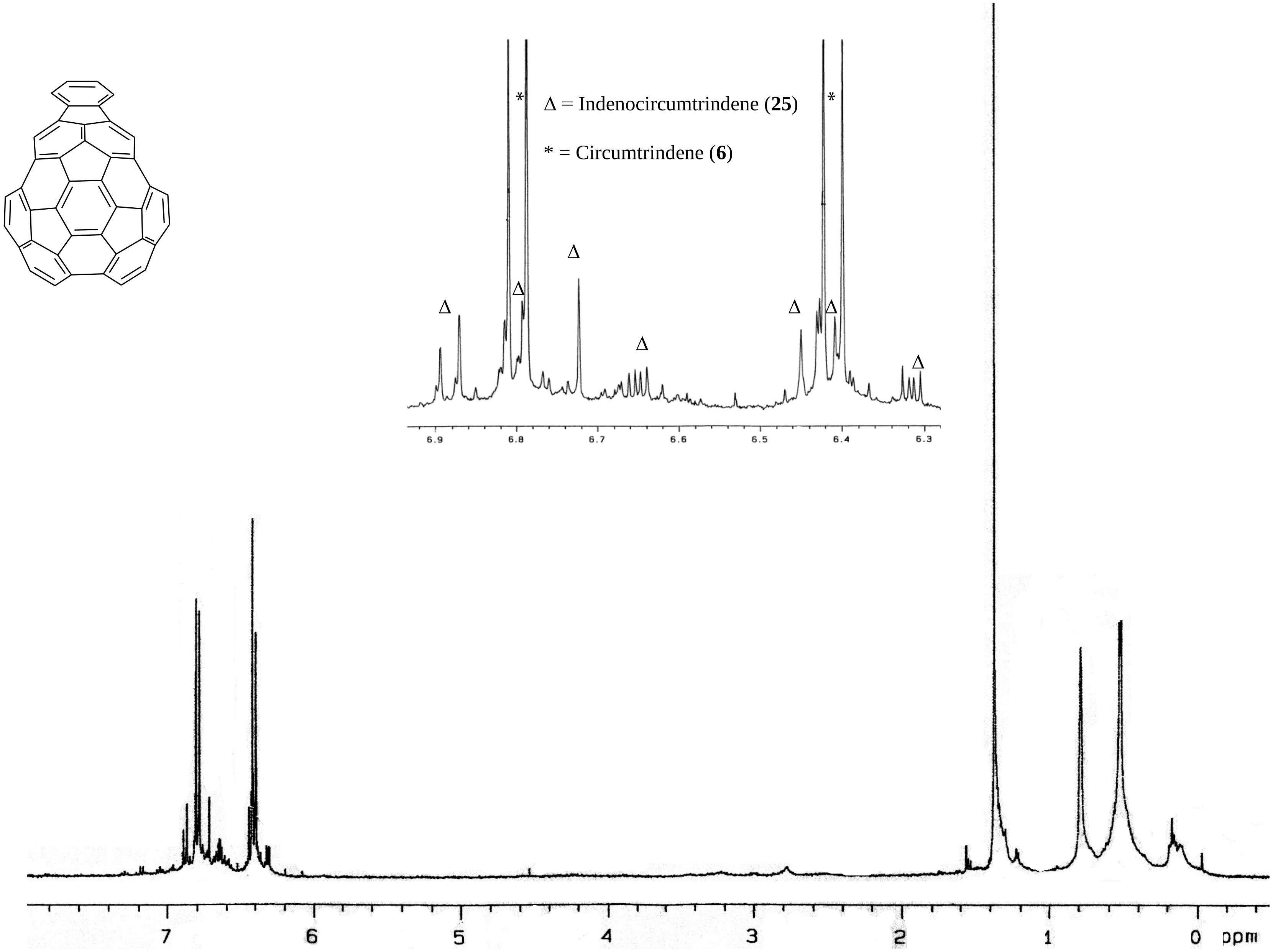
Δ = Indenocircumtrindene (**25**)
* = Circumtrindene (**6**)
ppm

1-(2,6-Dimethylphenyl)circumtrindene (30)

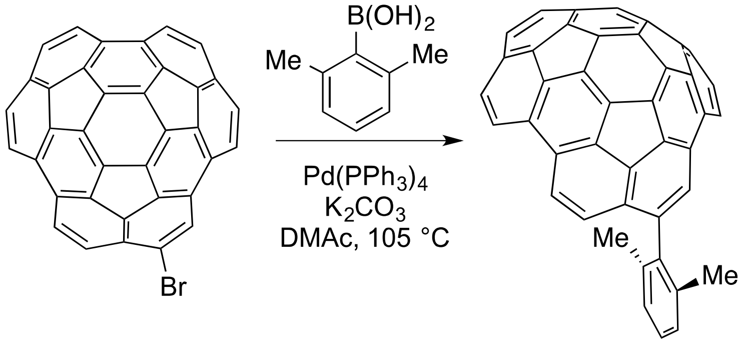

In a 25 mL Schlenk flask equipped with a magnetic stirring bar were mixed approximately 3 mg (0.0057 mmol) 1-bromocircumtrindene (**28**), 10 mg (0.067 mmol) 2,6-dimethylphenylboronic acid, 1 mg (0.001 mmol) tetrakis(triphenylphosphine) palladium, 25 mg (0.18 mmol) potassium carbonate, and 1 mL dimethylacetamide (DMAc). The reaction vessel was purged with nitrogen and lowered into a preheated wax bath at 105 °C. After stirring overnight, the reaction was quenched with a 10% aqueous hydrochloric acid solution and extracted into dichloromethane. Washing with saturated aqueous sodium bicarbonate, then saturated aqueous sodium chloride followed by drying over magnesium sulfate and removal of the solvent under reduced pressure resulted in crude 1-(2,6-dimethylphenyl)circumtrindene (**30**) as a yellow solid. Separation was achieved on an alumina preparative TLC plate with 5% ethyl acetate in hexanes. Of the two yellow bands that moved up the plate, the top one was **30**, and the bottom one was a small amount of circumtrindene. Yield: 70–80%. ^{1}H NMR (400 MHz, $CDCl_3$): δ 7.66 (d, *J* = 9.2 Hz, 1H), 7.61 (d, *J* = 9.2 Hz, 1H), 7.57 (d, *J* = 9.2 Hz, 1H), 7.50 (d, *J* = 9.2 Hz, 1H), 7.48 (d, *J* = 9.2 Hz, 1H), 7.41 (s, 1H), 7.27 (d, *J* = 9.2 Hz, 1H), 7.22 (d, *J* = 8.8 Hz, 1H), 7.19 (d, *J* = 9.2 Hz, 1H), 7.17 (d, *J* = 5.6 Hz, 2H), 7.15 (d, *J* = 8.8 Hz, 1H), 6.97 (t, *J* = 5.2 Hz, 1H), 6.74 (d, *J* = 9.2 Hz, 1H), 2.31 (s, 3H), 1.25 (s, 3H). ^{1}H NMR (400 MHz, CD_2Cl_2): δ 7.66 (d, *J* = 9.2 Hz,

1H), 7.65 (d, *J* = 9.2 Hz, 1H), 7.60 (d, *J* = 9.2 Hz, 1H), 7.55 (d, *J* = 8.8 Hz, 1H), 7.51 (d, *J* = 9.2 Hz, 1H), 7.41 (s, 1H), 7.26 (d, *J* = 9.2 Hz, 1H), 7.25 (d, *J* = 9.2 Hz, 1H), 7.23 (d, *J* = 9.2 Hz, 1H), 7.22 (d, *J* = 8.8 Hz, 1H), 7.16 (d, *J* = 5.2 Hz, 2H), 6.95 (t, *J* = 4.8 Hz, 1H), 6.71 (d, *J* = 9.2 Hz, 1H), 2.29 (s, 3H), 1.12 (s, 3H). ^{13}C NMR (125 MHz, $CDCl_3$): δ 152.42, 152.34, 152.28, 152.00, 151.98, 151.93, 139.80, 139.72, 139.54, 139.43, 139.42, 138.99, 138.57, 138.06, 137.75, 137.11, 137.08, 136.80, 136.22, 135.47, 135.30, 134.99, 134.94, 134.80, 134.56, 127.84, 127.79, 127.57, 127.53, 127.46, 127.24, 127.17, 126.88, 126.41, 126.25, 125.87, 125.84, 125.65, 125.40, 125.26, 124.85, 124.66. HRMS (EI, 70 eV) calculated for $C_{44}H_{20}$ (M^+): 548.1565, found: 548.1556.

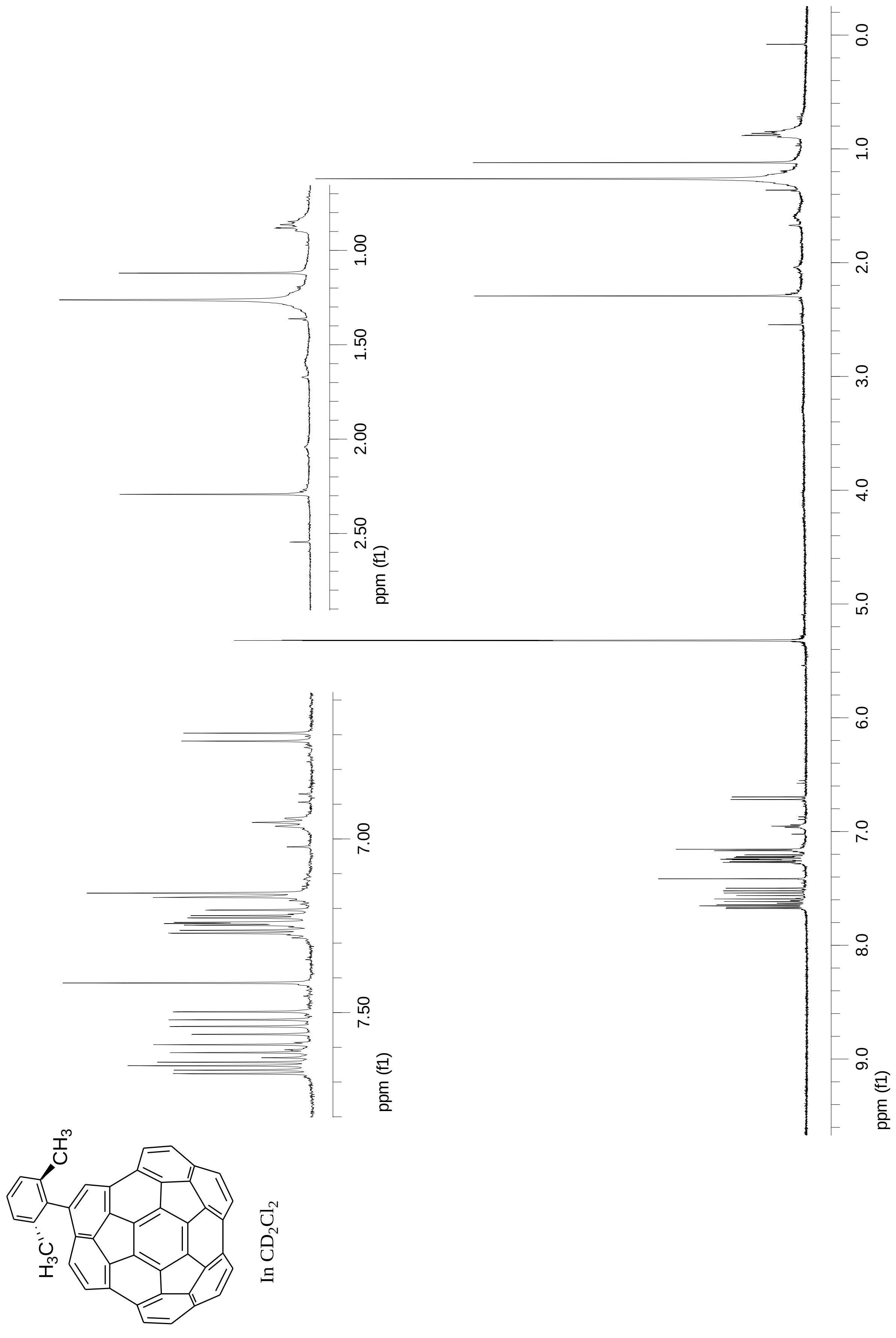

H_3C
CH_3
In CD_2Cl_2
ppm (f1)

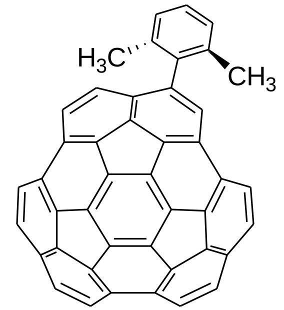

In $CDCl_3$

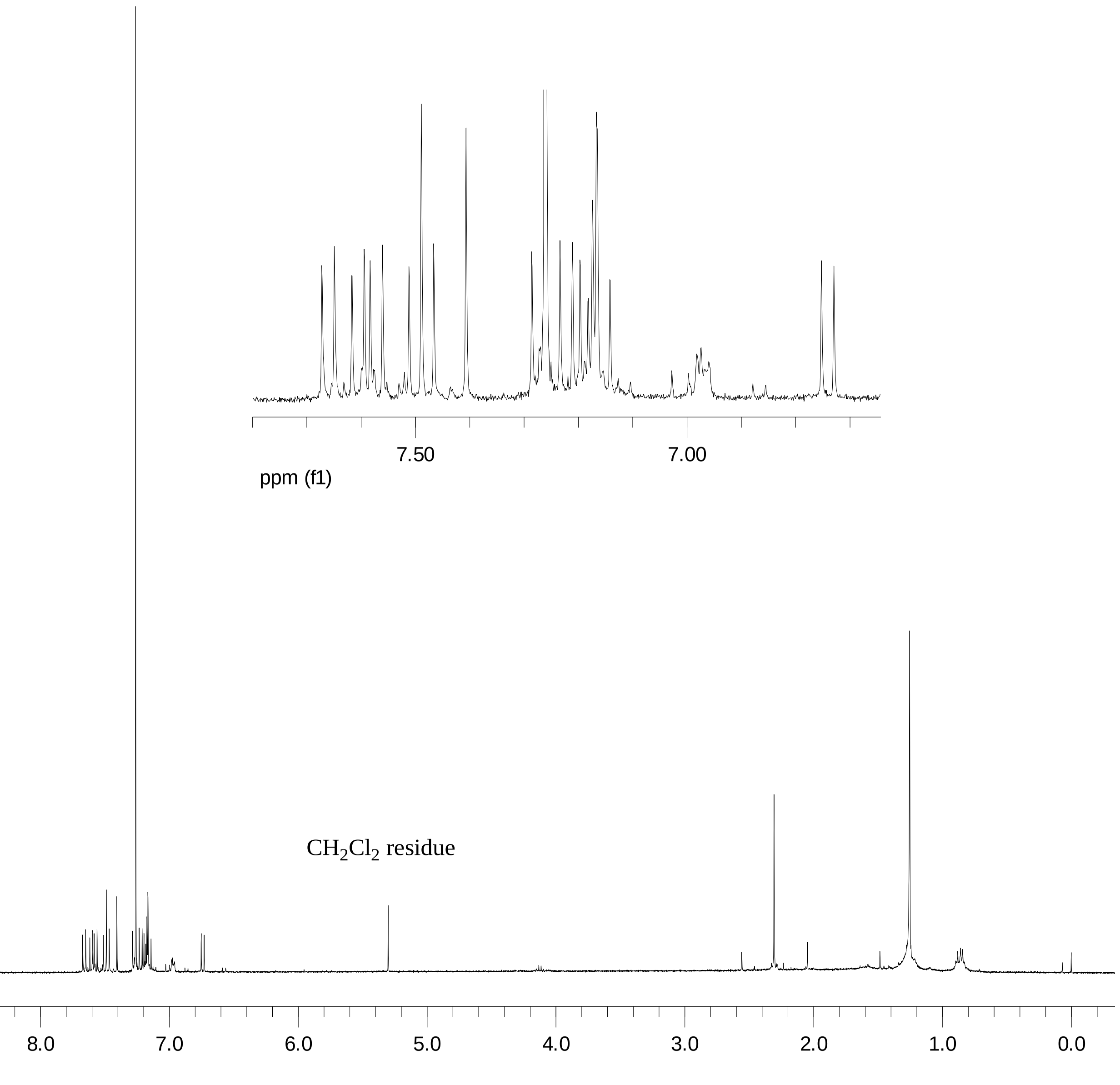

7.50
7.00
ppm (f1)
CH2Cl2 residue
10.0
9.0
8.0
7.0
6.0
5.0
4.0
3.0
2.0
1.0
0.0
ppm (f1)

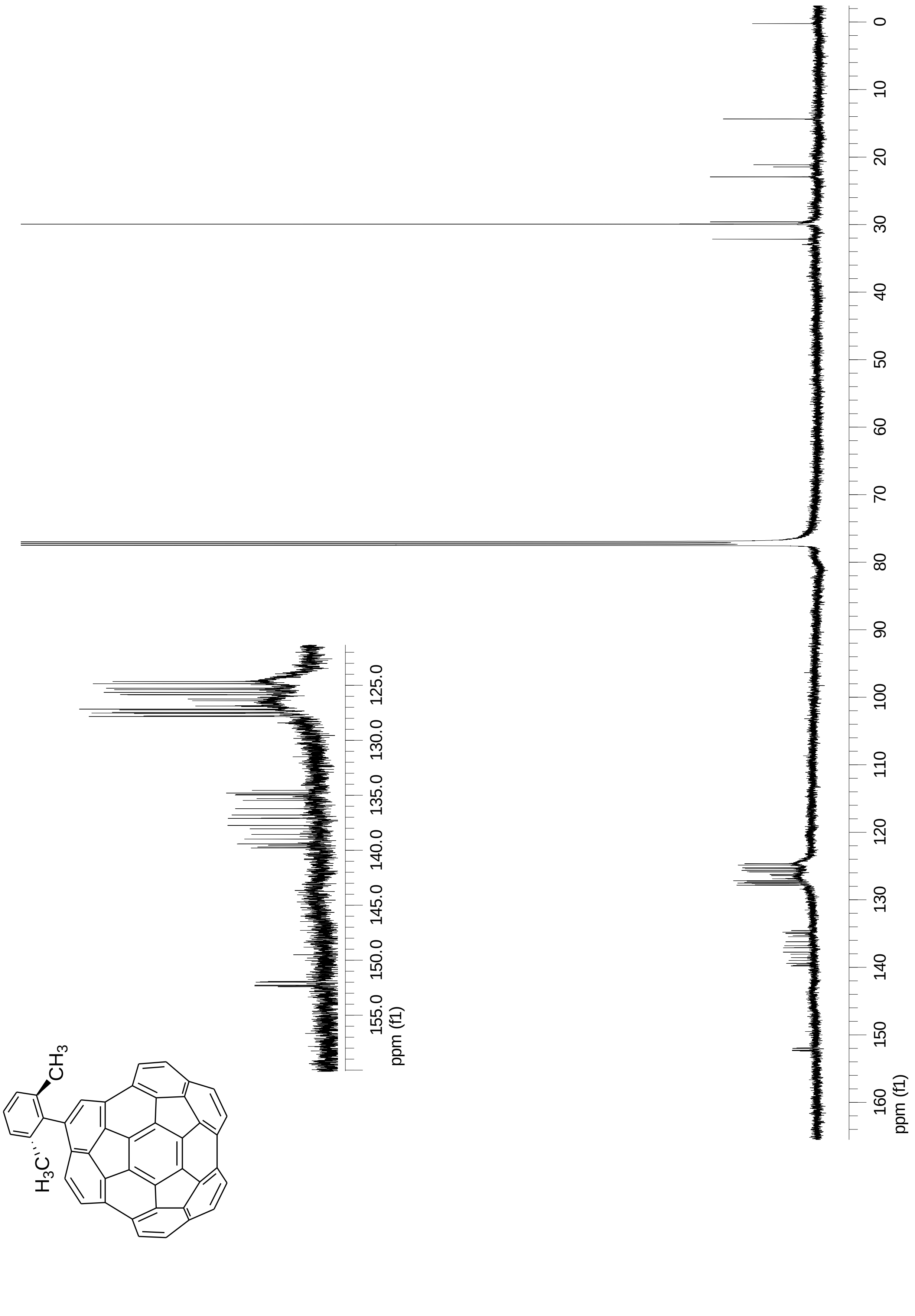

ppm (f1)
ppm (f1)

1-(2,6-Dimethylphenyl)naphthalene (31)[3]

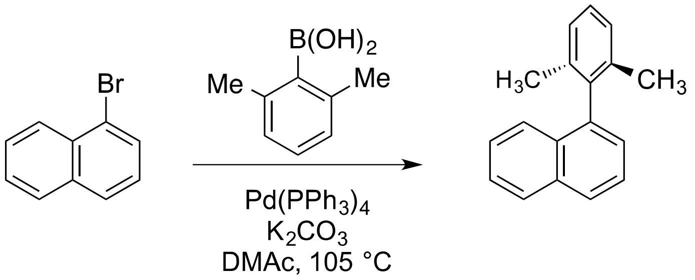

Into a 25 mL Schlenk flask, equipped with a magnetic stirring bar, were added 110 mg (0.53 mmol) 1-bromonaphthalene, 120 mg (0.80 mmol) 2,6-dimethylphenyl-boronic acid, 60 mg (0.052 mmol) tetrakis(triphenylphosphine)palladium, 365 mg (2.64 mmol) potassium carbonate, and 3 mL dimethylacetamide. The reaction mixture was lowered into a preheated wax bath at 105 °C and stirred overnight, with the temperature fluctuating between 105–110 °C. After cooling and quenching with 10% aqueous hydrochloric acid, the reaction mixture was extracted into dichloromethane, washed with saturated sodium bicarbonate and saturated sodium chloride. Drying over magnesium sulfate and removal of the solvent under reduced pressure gave 1-(2,6-dimethylphenyl)naphthalene (**31**) as a crude product. Preparative TLC on silica gel (hexanes) resulted in a clean sample suitable for NMR analysis in 80–90% yield (100–110 mg). ^{1}H NMR (400 MHz, $CDCl_3$): δ 7.91 (d, J = 8.4 Hz, 1H), 7.86 (d, J = 8.0 Hz, 1H), 7.54 (t, J = 8.0 Hz, 1H), 7.49-7.45 (m, 1H), 7.37-7.33 (m, 2H), 7.28-7.24 (m, 2H), 7.18 (d, J = 7.2 Hz, 2H), 1.90 (s, 6H). ^{13}C NMR (100 MHz, $CDCl_3$): δ 139.70, 138.82, 137.07 (2C), 133.82, 131.83, 128.37, 127.41, 127.35 (2C), 127.29, 126.53, 126.15, 125.87, 125.79, 125.48, 20.74 (2C). LRMS (EI, 70 eV) m/z (relative intensity): 232 (M^+, 100), 217 (81), 202 (69), 188 (22), 107 (16). HRMS (EI, 70 eV) calculated for $C_{18}H_{16}$ (M^+): 232.1252. Found: 232.1248.

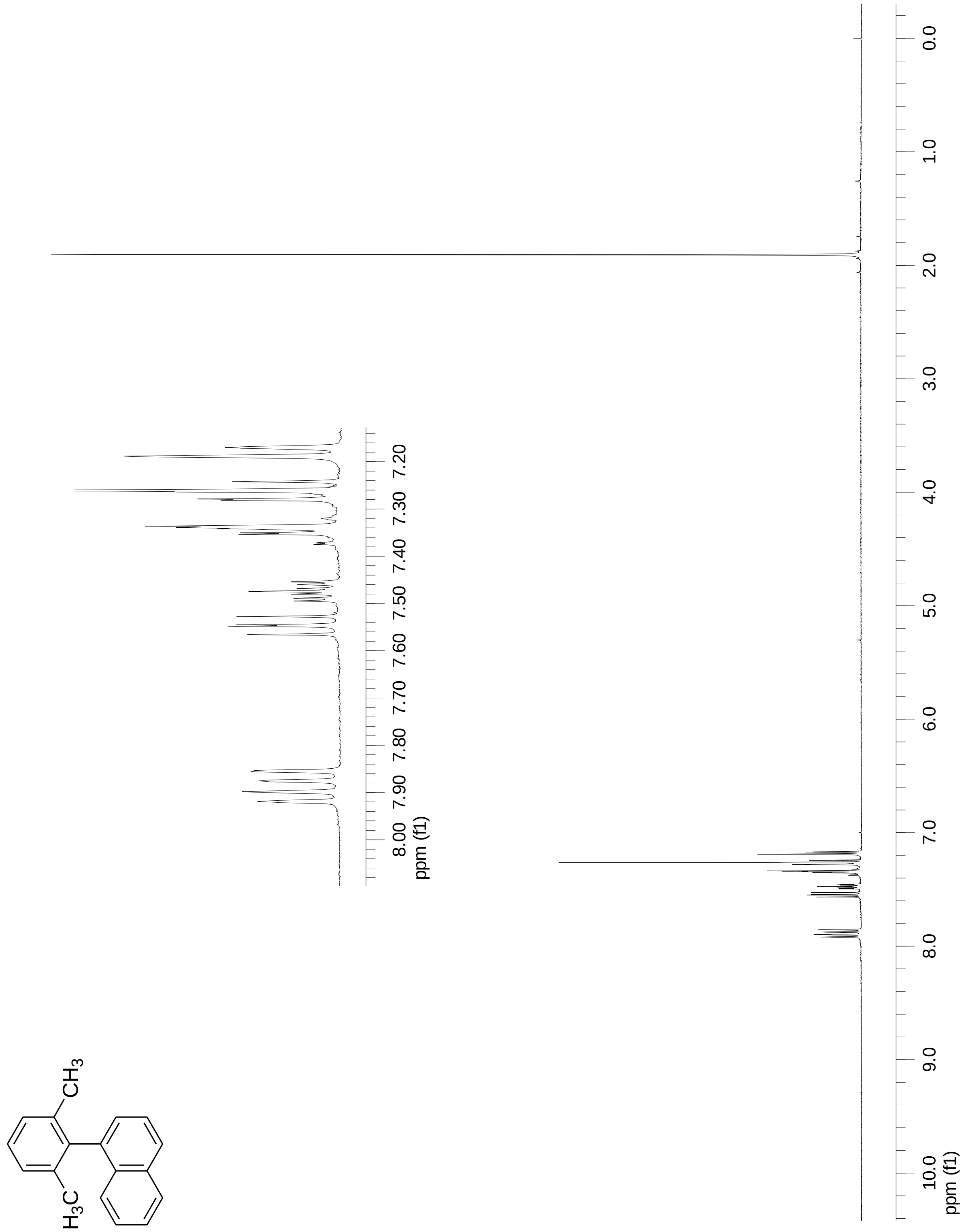
CH3
H3C
ppm (f1)
ppm (f1)

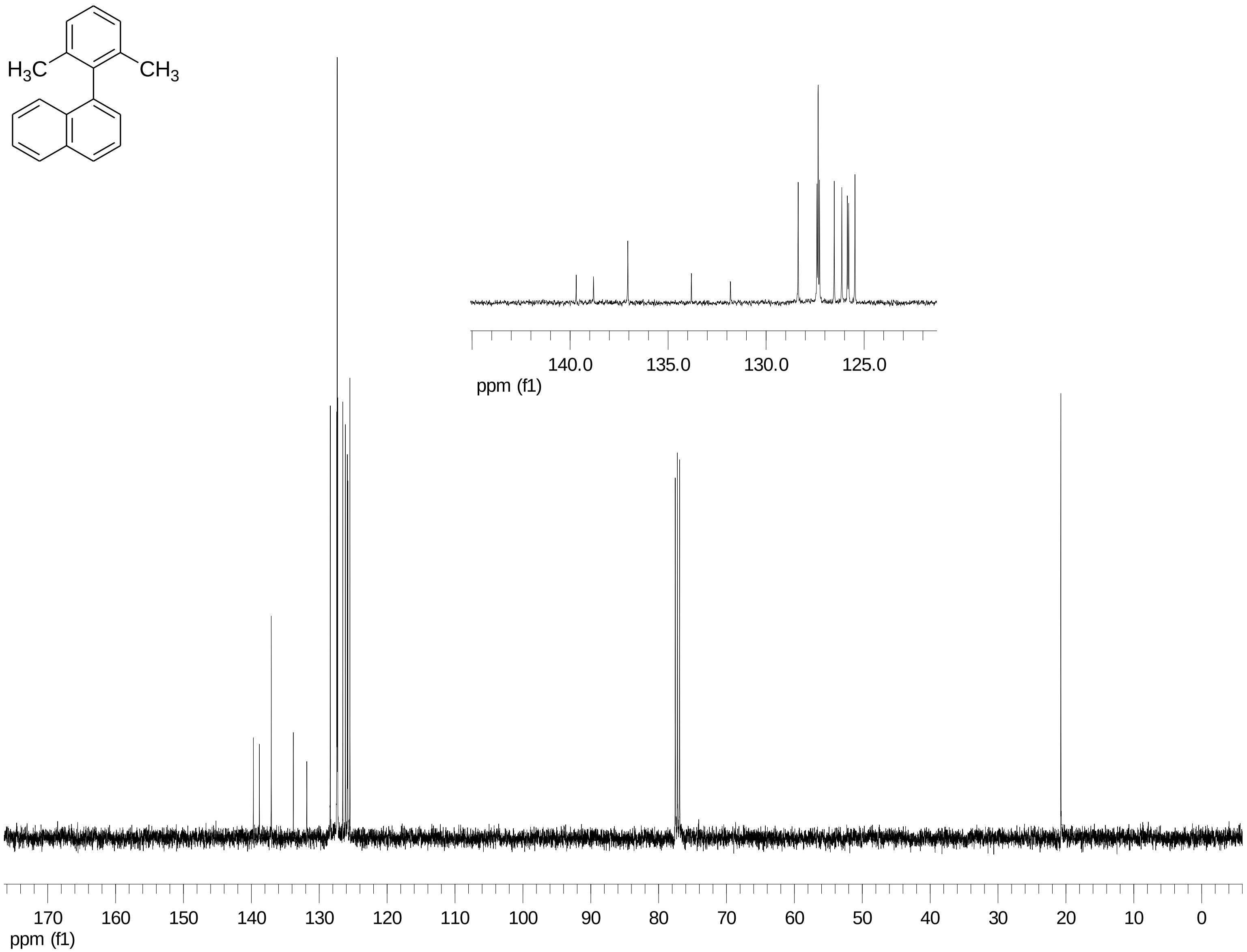

H_3C
CH_3
140.0
135.0
130.0
125.0
ppm (f1)
170
160
150
140
130
120
110
100
90
80
70
60
50
40
30
20
10
0
ppm (f1)

References

1 Still, W. C.; Kahn, M.; Mitra, A. *J. Org. Chem.* **1978**, *43*, 2923-2925.

2 Ansems, R. B. M.; Scott, L. T. *J. Am. Chem. Soc.* **2000,** *122*, 2719-2724.

3 First synthesis: Lipshutz, B. H.; Siegmann, K.; Garcia, E.; Kayser, F. *J. Am. Chem. Soc.* **1993,** *115*, 9276-82.
